# Combination Treatment With Inhibitors of ERK and Autophagy Enhances Antitumor Activity of Betulinic Acid in Non–small-Cell Lung Cancer *In Vivo* and *In Vitro*

**DOI:** 10.3389/fphar.2021.684243

**Published:** 2021-06-29

**Authors:** Chao-Yue Sun, Di Cao, Qian-Nan Ren, Shan-Shan Zhang, Ning-Ning Zhou, Shi-Juan Mai, Bing Feng, Hui-Yun Wang

**Affiliations:** ^1^ State Key Laboratory of Oncology in South China, Collaborative Innovation Center for Cancer Medicine, Sun Yat-Sen University Cancer Center, Guangzhou, China; ^2^ Department of Medical Oncology, Sun Yat-Sen University Cancer Center, Guangzhou, China; ^3^ Guangdong Provincial Hospital of Chinese Medicine, The Second Clinical College of Guangzhou University of Chinese Medicine, Guangzhou University of Chinese Medicine, Guangzhou, China

**Keywords:** lung cancer, betulinic acid, autophagy, apoptosis, ERK, combination

## Abstract

Aberrant activation of the Ras–ERK signaling pathway drives many important cancer phenotypes, and several inhibitors targeting such pathways are under investigation and/or approved by the FDA as single- or multi-agent therapy for patients with melanoma and non–small-cell lung cancer (NSCLC). Here, we show that betulinic acid (BA), a natural pentacyclic triterpenoid, inhibits cell proliferation, and induces apoptosis and protective autophagy in NSCLC cells. Thus, the cancer cell killing activity of BA is enhanced by autophagy inhibition. Mitogen-activated protein kinases, and especially ERK that facilitates cancer cell survival, are also activated by BA treatment. As such, in the presence of ERK inhibitors (ERKi), lung cancer cells are much more sensitive to BA. However, the dual treatment of BA and ERKi results in increased protective autophagy and AKT phosphorylation. Accordingly, inhibition of AKT has a highly synergistic anticancer effect with co-treatment of BA and ERKi. Notably, autophagy inhibition by hydroxychloroquine (HCQ) increases the response of lung cancer cells to BA in combination with ERKi. *In vivo*, the three-drug combination (BA, ERKi, and HCQ), resulted in superior therapeutic efficacy than single or dual treatments in the xenograft mouse model. Thus, our study provides a combined therapy strategy that is a highly effective treatment for patients with NSCLC.

## Introduction

Although lung cancer incidence continues to decline in some countries, it remains the leading cause of cancer-related death in the world (Melosky et al., 2018; Siegel et al., 2018; Siegel et al., 2020), in which approximately 84% of lung cancers are non–small-cell lung cancer (NSCLC), and 70% of newly diagnosed NSCLC is in an advanced or metastatic stage (Kelly et al., 2018; Reinmuth, 2018). Although there are many therapeutic options for NSCLC patients, the 5-year survival rate of NSCLC remains less than 15% in China (Macdonagh et al., 2018). Drug combination is a better strategy for cancer treatment than monotherapy. Thus, exploring effective strategies especially multiple drug combinations is a challenge for clinical management and improving survival in NSCLC patients.

Autophagy is an highly evolutionarily conserved cellular process that sequesters damaged, dysfunctional proteins or organelles in autophagosomes and delivers these materials to lysosomes for degradation (Doherty and Baehrecke, 2018). Autophagy is considered as a double-edged sword in cancer biology, depending on the context. The basal level of autophagy, under physiological conditions, enables the disposal of abnormal structures to maintain cellular homeostasis (Dower et al., 2018). However, dysregulated or excessive autophagy is implicated in various pathological conditions, especially for cancer (Nyfeler and Eng, 2016). In most cases, autophagy has emerged as a pro-survival mechanism in response to adverse environmental conditions or therapeutic challenges (Das et al., 2018), indicating that autophagy can confer drug resistance. Accordingly, inhibition of autophagy has the potential to kill cancer cells or improve anticancer therapeutics. Paradoxically, autophagy can induce cell death, termed autophagic cell death, and thus, such autophagy inducers have potential to kill cancer cells (Fulda and Kogel, 2015). Therefore, pharmacological manipulation of autophagy, based on the differential cell fate, has the potential to enhance cancer therapy.

The extracellular signal–regulated kinases (ERKs) is frequently hyperactivated in many types of cancer, which is usually induced by activating mutations of the K-Ras that accounts for approximately 25% of NSCLC patients (Chatterjee et al., 2017). ERK can activate a series of transcription factors that orchestrate a complex cellular response, which is often considered as pro-proliferative (Brandt et al., 2019). To date, direct inhibition of K-Ras has not yet been successful in clinical therapy, whereas inhibitors targeting ERK, such as binimetinib and trametinib, are approved to treat melanoma (Roskoski, 2020) and are under clinical trial for treatment of lung cancer (Heigener et al., 2015). Despite this, response rates of these ERK inhibitors (ERKi) are highly variable in cancer patients, and acquired resistance limits their efficacy. In this regard, increased autophagy has been proven to contribute to drug resistance. For example, ERK inhibition by trametinib elicits autophagic flux that protects pancreatic ductal adenocarcinoma (PDA) from cell death, and dual inhibition of ERK and autophagy has a synergistic antiproliferative activity (Bryant et al., 2019; Kinsey et al., 2019). Moreover, resistance to ERKi occurs by compensatory activation of the PI3K–AKT pathway that regulates various cell processes, such as proliferation and metastasis (Mendoza et al., 2011). Accordingly, combination of drugs directed against ERK and another target plays an essential role in the future development of new therapeutic strategy.

Phytochemicals are biologically active compounds isolated from plants or herbs, which are recognized as potent anticarcinogenic agents (Yin et al., 2017; Ng et al., 2018; Chen et al., 2020). Betulinic acid (BA, (3β)-3-hydroxyl-lup-20 (29)-en-28-oic acid, the chemical structure of BA shown in Figure 9G) is a naturally lupane-structured pentacyclic triterpenoid that is mainly widespread in white-barked birch trees (Zhang et al., 2015). BA has been demonstrated to possess cytotoxic effect toward multiple types of tumors, including breast cancer, lung cancer, prostate cancer, gastric cancer, and colon cancer (Luo et al., 2016; Cai et al., 2018). Mechanistic studies reveal that BA inhibits cancer cell proliferation by suppressing specificity protein transcription factors (Sp), transcription factor nuclear factor kappa B (NF-κB), vascular endothelial growth factor (VEGF), and topoisomerase (Ng et al., 2010; Parrondo et al., 2010). In addition, BA induces cytoprotective autophagy in colorectal cancer cells by inhibiting AKT-mTOR signaling, and autophagy blockade augments apoptotic cell death (Wang et al., 2017).

In the present study, we investigated the effect of BA alone or in combination with ERKi on cell proliferation of NSCLC cells, and uncovered the role of autophagy in these treatments. We also aimed to elucidate the underlying molecular mechanism of these agents. Our results clearly demonstrated that BA in combination with ERKi plus autophagy inhibition is a reasonable therapeutic strategy for NSCLC.

## Materials and Methods

### Reagents and Antibodies

BA (purity >97%) was purchased from Aladdin (Shanghai, China) and dissolved in DMSO. A stock solution of 10 mM BA was prepared and stored at −20°C. SP600125 (JNK inhibitor), SB203580 (p38 inhibitor), U0126 (ERK inhibitor), MK2206 (AKT inhibitor), hydroxychloroquine (HCQ, autophagy inhibitor), trametinib, and MEK162 (ERK inhibitor) were obtained from Selleck Chemicals (Houston, United States). Primary antibodies follow: β-actin (CST, 4970S), GAPDH (Abcam, ab181602), LC3 (CST, 12741S), ATG5 (CST, 12994S), p38 (Santa Cruz, sc-271120), p-p38 (Santa Cruz, sc-166182), ERK1/2 (Proteintech, 16443-1-AP), p-ERK1/2 (ST, 4370T), JNK (Santa Cruz, sc-7345), p-JNK (CST, 4668T), PARP (CST, 9542S), cleaved PARP (CST, 5625S), AKT (CST, 4691S), p-AKT (CST, 4060P), CDK2 (Abcam, ab205718), CDK4 (Abcam, ab108357), and cyclin D1 (Abcam, ab134175). Horseradish peroxidase (HRP)-conjugated secondary antibodies were supplied by Cell Signaling Technology.

### Cell Lines and Cell Culture

The human NSCLC cell lines, including A549, H1650, and H1975, were provided by Shanghai Life Sciences Research Institute Cell Resources Center. All cell lines were cultured in RPMI-1640 medium containing 10% FBS (Gibco Laboratories, Grand Island, United States) and were maintained at 37°C in a standard incubator with a 5% CO_2_ supplement.

### Cell Viability Assays

The MTT method was employed to determine the cell viability. In brief, cells were plated into 96-well plates at a density of 4×10^3^ cells/well. After incubation overnight, cells were exposed to BA alone, or in combination with other chemicals at the indicated concentrations for 24 and 48 h. After treatment, each well was added with 20 μL MTT (5 mg/ml) solution, followed by addition with 150 μL DMSO. The absorbance at 490 nm of the plate was measured by a Multiskan Ascent Revelation Plate Reader (Thermo, United States). The synergistic effect of combined treatments was evaluated by CompuSyn software package (Biosoft, Cambridge, UK). The combination index (CI) values of 0.9–1.1 indicate additive effect, and CI values of 0.1–0.9 indicate synergism (Adhireksan et al., 2017).

### siRNA Transfection

Briefly, cells were plated into 6-well plates and cultured to approximately 50–60% confluency. Cells were transfected with siRNAs targeting ATG5 (5′GCA​ACT​CTG​GAT​GGG​ATT​G 3′, 5′CAT​CTG​AGC​TAC​CCG​GAT​A 3′) using Lipofectamine 3000, according to the manufacturer’s protocols. Scrambled siRNA was used as a negative control.

### Cell Cycle Analysis

Cells were incubated in 6-well plates (2.0 × 10^5^ cells/well) and treated with BA alone, or in combination with trametinib for 48 h. After treatment, cells were harvested, washed with PBS, and fixed with cold 75% ethyl alcohol at 4 °C overnight. Then, cells were stained with 1ml DNA staining solution (MultiSciences Biotech Co., Ltd.) at room temperature for 30 min. The cell cycle was performed by a flow cytometer (BD Biosciences, San Jose, CA).

### Apoptosis Analysis by Flow Cytometry and Hoechst Staining

Cells were seeded in 6-well plates (2.0 × 10^5^ cells/well) and treated with BA alone, or in combination with other inhibitors for 48 h. After treatment, cells were harvested and washed with ice-cold PBS. Then, cells were double-stained with 5 μL Annexin V-FITC and 10 μL PI solution from the Annexin V-FITC/PI Apoptosis Kit (MultiSciences Biotech Co., Ltd.) for 5 min at room temperature in the dark. The apoptosis rate was immediately analyzed using a flow cytometer (BD Biosciences, San Jose, CA). For Hoechst staining, cells were stained with 5μL Hoechst 33342 (5 mg/ml) for 5 min in the dark; then cells were immediately imaged using a fluorescent microscope (Nikon, Japan).

### Western Blot Analysis

Cells were lysed in RIPA lysis buffer (CWBio Co., Ltd.) containing 1% protease and phosphatase inhibitors (Thermo Scientific). Protein concentration was measured using a BCA Protein Assay Kit (Thermo Scientific). Samples (20 μg) were separated by electrophoresis on 8–15% SDS-PAGE gel, and then transferred onto PVDF membranes (0.45 μm; Merck Millipore) using a wet transfer system (Bio-Rad, United States). After blocking with 5% skimmed milk for 1 h at room temperature, the membranes were incubated overnight with primary antibodies at 4°C, followed by secondary antibodies for another 1 h. Blots were detected by the enhanced chemiluminescence (ECL) system (Millipore, United States).

### mRFP-GFP-LC3 Adenovirus Transfection

mRFP-GFP-LC3 adenovirus was purchased from Vigene Biosciences (Shandong, China, NM022818). Cells were seeded in 96-well plates, and then transfected with adenovirus expressing mRFP-GFP-LC3 together with the ADV-HR (Vigene Biosciences, FH880805), according to the manufacturer’s protocol. After transfection, the medium was replaced with fresh culture medium, and cells were treated with BA for 48 h. Then cells were imaged using a fluorescence microscope (Nikon, Japan).

### Transmission Electron Microscopy (TEM)

Cells were washed in PBS and fixed in 2.5% glutaraldehyde on ice for 4 h. Then cells were postfixed in 1% osmic acid for 2 h and dehydrated in a series of gradient ethanol and acetone. Samples were then embedded in araldite, and ultrathin sections (50–60 nm) were cut. Sections were stained with uranyl acetate and lead citrate, and images were obtained using an electron microscope (Hitachi H-7650, Tokyo, Japan).

### *In Vivo* Xenograft Model

The mouse experiments were in strict compliance with protocols approved by the Animal Care and Use Committee of Sun Yat-Sen University Cancer Center (protocol ID: L102012019110A). Female 4- to 6-week-old BALB/c nude mice were provided by Guangdong Medical Laboratory Animal Center (Foshan, China), which were subcutaneously implanted with 4 × 10^6^ A549 cells, into the right flank. When tumor volume became palpable, approximately 100 mm^3^, 56 mice were randomized to eight treatment groups: the first group, control; the second group, BA (20 mg/kg); the third group, trametinib (1 mg/kg); the fourth group, HCQ (60 mg/kg); the fifth group, BA + trametinib; the sixth group, BA + HCQ; the seventh group, trametinib + HCQ; and the eighth group, BA+ trametinib + HCQ. Trametinib and BA were dissolved in DMSO and diluted in water with 0.5% carboxymethylcellulose (Meilunbio) and 0.2% Tween-80 (Sigma), while HCQ was dissolved in water. All drugs were administered every day by oral gavage (0.1 ml per animal), continuously for 15 days. Mice weight and tumor volume were measured every three days. After treatment, mice were euthanized and tumors were harvested.

### Statistical Analysis

Statistical analyses were performed using SPSS software (version 17.0; Chicago, United States), and all values are presented as mean ± SEM. Student’s t-test was employed to compare values between two groups, while one-way analysis of variance (ANOVA) was used to compare values between multiple groups. *p* values < 0.05 were considered as statistically significant. All experiments were repeated more than three times.

## Results

### BA Inhibits the Proliferation of NSCLC Cells *via* Inducing Apoptosis

**FIGURE 1:**
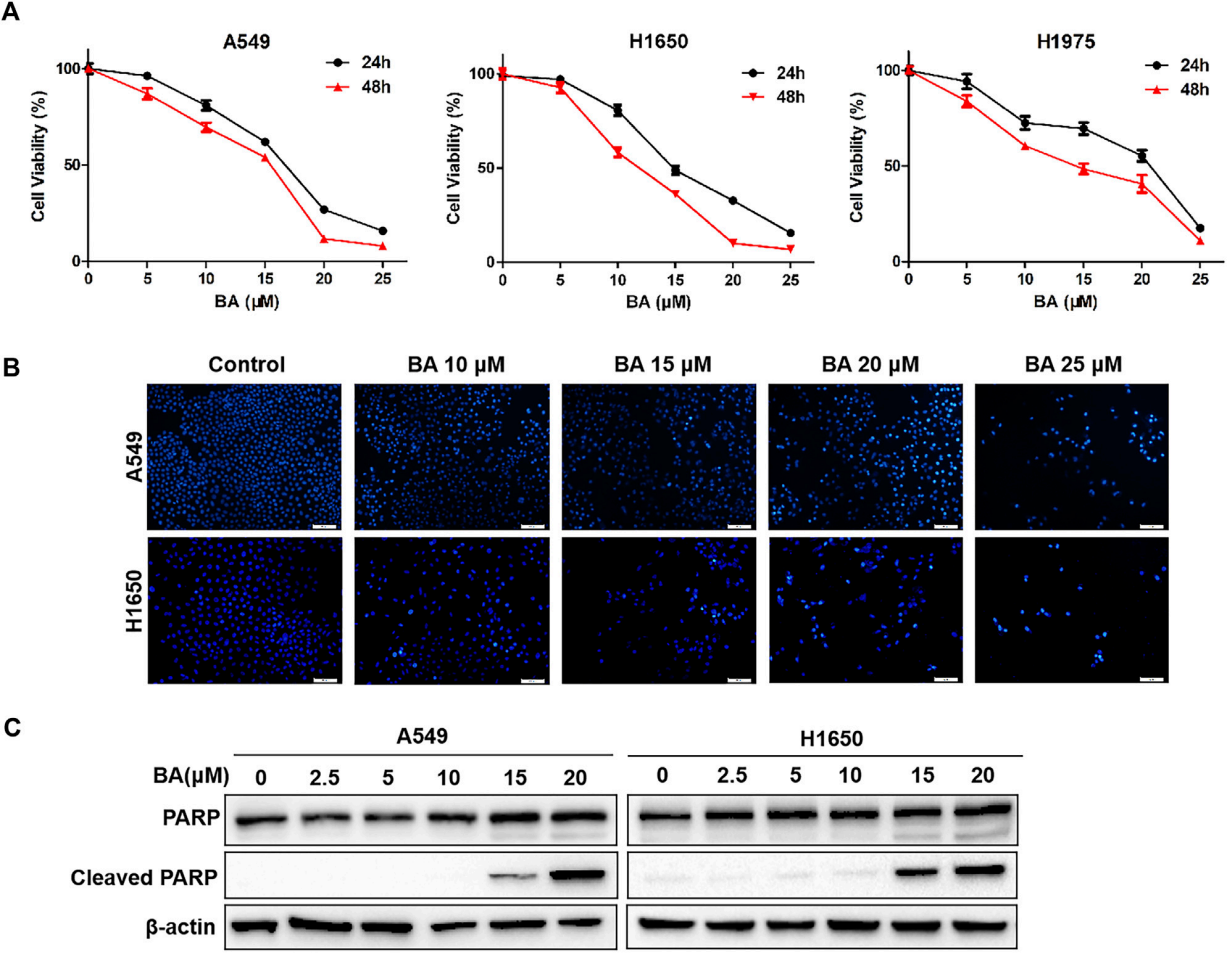
BA inhibits the proliferation of NSCLC cells by inducing apoptosis. **(A)** A549, H1650, and H1975 cells were treated with increasing concentrations of BA for 24 and 48 h, and the cell viability was measured by the MTT assay. **(B)** A549 and H1650 cells were exposed to increased concentrations of BA for 24 h, and cell apoptosis was assessed by Hoechst 33342 staining. **(C)** A549 and H1650 cells were treated with BA for 24 h, and the levels of PARP and cleaved PARP were detected by Western blots. β-actin was used as a loading control. Data are expressed as mean ± SEM.

To confirm the potential cytotoxicity of BA on NSCLC cells, A549, H1650, and H1975 were treated with increasing concentrations of BA for 24 and 48 h, and the MTT assay was performed to measure cell viability. As shown in Figure 1A, BA treatment suppressed the growth of NSCLC cells in a dose- and time-dependent manner. The IC_50_ values, following 48 h treatment, were 15.67, 11.82, and 14.74 μM for A549, H1650, and H1975 cells, respectively. Thus, BA inhibited the proliferation of NSCLC cells, which is concordant with the reported results (Kutkowska et al., 2017).

Reports indicate that BA can induce apoptosis in human cancers (Kumar et al., 2018). Thus, we wonder whether BA inhibits NSCLC cells by inducing apoptosis. To this end, we treated NSCLC cells with increasing concentrations of BA and detected these cells using Hoechst 33343 staining. After treatment for 48 h, the apoptosis rates of A549 cells and H1650 cells were increased, as shown in Hoechst staining (Figure 1B), exhibiting that BA indeed induced apoptosis in NSCLC cells. In addition, we measured the apoptotic markers, PARP, and cleaved PARP, using Western blots. As expected, results revealed that the expression of cleaved PARP was elevated in A549 and H1650 cells treated with BA (Figure 1C). These results indicate that BA-induced apoptosis mediates inhibition of proliferation in NSCLC cells.

### BA-Induced Autophagy Antagonizes Apoptosis in NSCLC Cells

**FIGURE 2:**
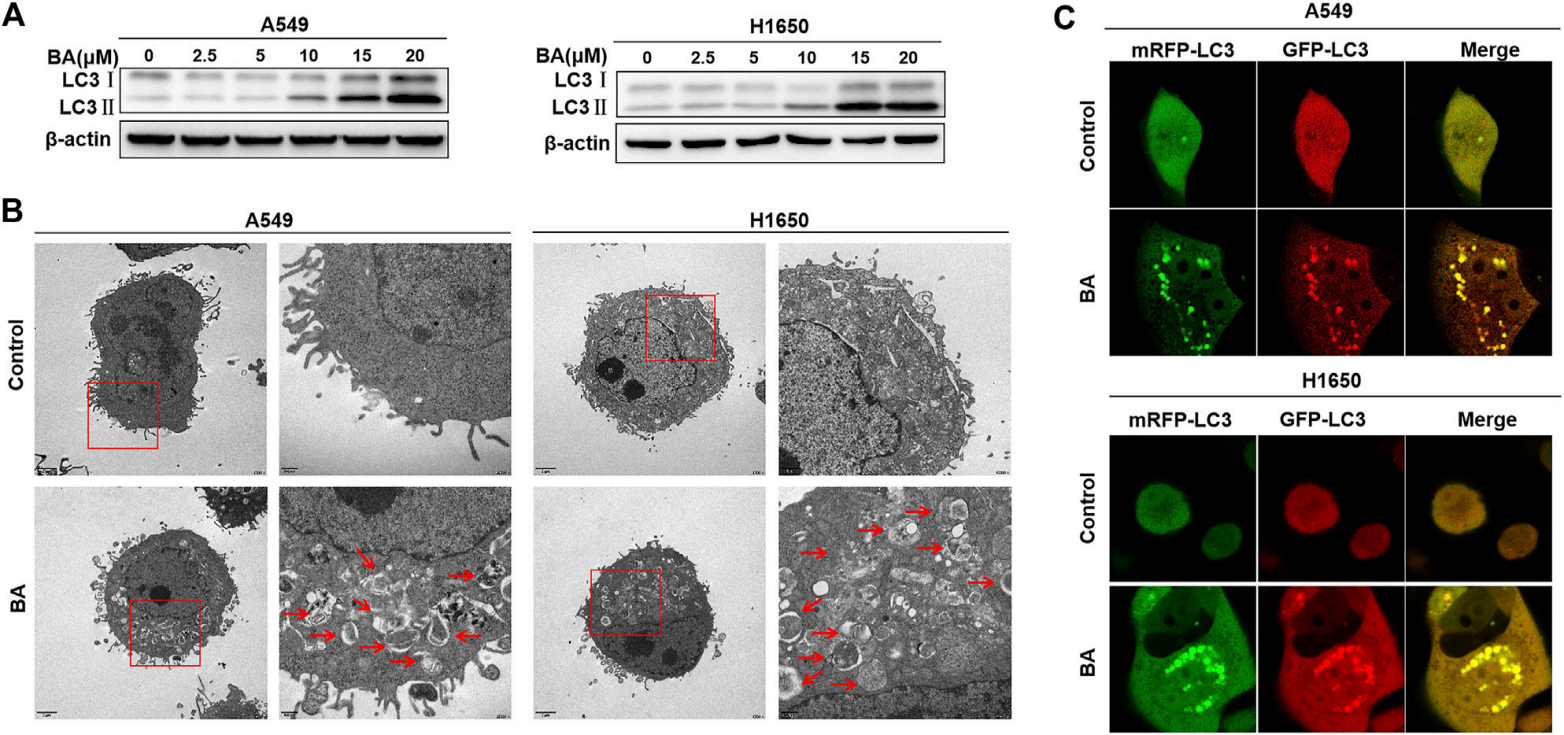
BA induces autophagy in NSCLC cells. **(A)** A549 and H1650 cells were treated with the indicated concentrations of BA for 48 h, and the expression of autophagy-related protein LC3 was analyzed by Western blots. **(B)** A549 and H1650 cells were treated with 15 μM BA for 48 h, and the autophagic vacuoles were observed using transmission electron microscopy. **(C)** A549 and H1650 cells were transfected with adenovirus expressing mRFP-GFP-LC3, then treated with 15 μM BA for 48 h, and representative images were obtained using a confocal microscope.

It has been reported that BA also can induce cell autophagy, which results in either cell death or survival (Li et al., 2019; Chen et al., 2020). Increasing evidence highlights that cancer cells have the ability to harness autophagy to thrive (Galluzzi et al., 2017). Here, we first investigated whether BA can induce autophagy in NSCLC cells, and then asked if the activated autophagy leads to cell survival. Usually, the increased LC3-II turnover is a key indicator of autophagy induction, in which the cytosolic form of LC3-I is converted into autophagosome-bound lipidated LC3-II, representing the formation of autophagosome (Marcelin et al., 2020). Thus, the increased level of the LC3-II or LC3-II/LC3-I ratio indicates the augmentation of autophagy. As shown in Figure 2A, BA effectively enhanced the expression of LC3-II in a dose-dependent manner in A549 and H1650 cells, suggesting that BA treatment can induce autophagy in NSCLC cells. Furthermore, ultrastructural analysis by TEM showed that BA increased the number of autophagic vacuoles per cell in A549 and H1650 cells when compared with the control cells (Figure 2B). To further confirm that the vacuoles in the BA-treated NSCLC cells are autophagosome, the mRFP-GFP-LC3 adenovirus was transfected into A549 and H1650 cells. As expected, many fluorescent dots of LC3 were observed in A549 and H1650 cells treated with BA compared with the control cells (Figure 2C), implying the autophagosome formation in NSCLC cells. These results consistently indicate that BA provoked autophagy in NSCLC cells.

**FIGURE 3:**
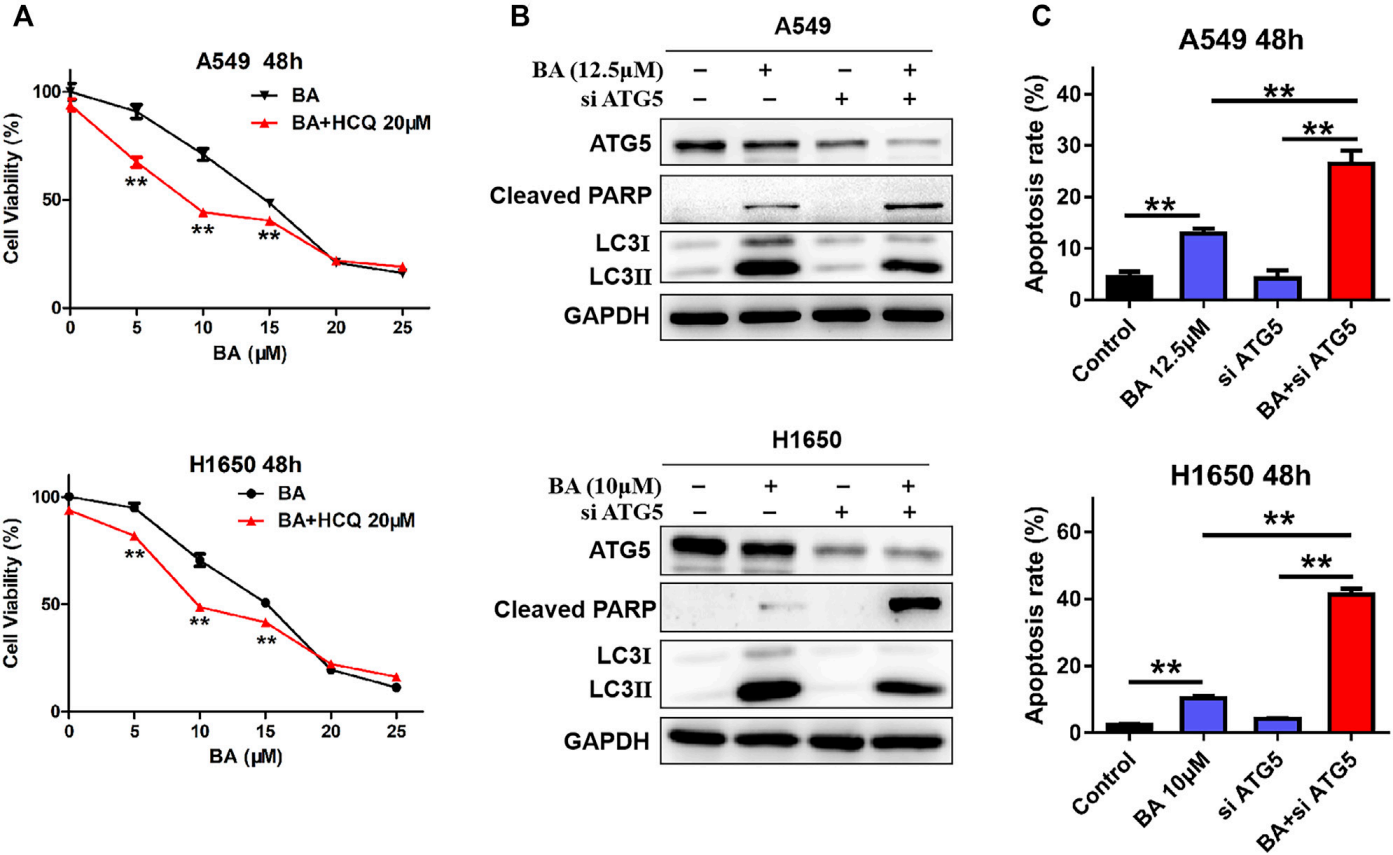
BA-induced autophagy promotes cell survival. **(A)** A549 and H1650 cells were treated with increasing concentrations of BA alone, or in combination with autophagy inhibitor HCQ for 48 h, and cell viability was measured by the MTT assay. **(B)** A549 and H1650 cells were transfected with siRNA against ATG5, followed by treatment with BA, and the expressions of LC3, ATG5, and cleaved PARP were analyzed by Western blots. **(C)** A549 and H1650 cells were transfected with siRNA of ATG5, following by BA treatment, and apoptosis was assessed using flow cytometry. **p < 0.01.

Then, we elucidated the role (promoting cell death or survival) of BA-induced autophagy in NSCLC cells. Hydroxychloroquine (HCQ), an autophagy inhibitor that blocks the fusion of autophagosomes with lysosomes, leading to LC-3II accumulation (Collins et al., 2018), was used to co-treat NSCLC cells with BA. As shown in Figure 3A, a significant increase of the percentage of dead cells was observed when A549 and H1650 cells were co-treated with 20 μM HCQ and 5, 10, or 15 μM BA, indicating that the autophagy plays an antagonistic role to BA-induced cell death, and autophagy inhibition sensitizes NSCLC cells to BA treatment. To further ascertain the protective effect of BA-induced autophagy in NSCLC cells, NSCLC cells were transfected with a specific siRNA against autophagy-related gene 5 (ATG5) that is required for the initiation of autophagosome formation. As shown in Figure 3B, A549 and H1650 cells with downregulation of ATG5 and BA treatment exhibited a notable decrease in the LC3II level and increase in the cleaved PARP level when compared with the BA-treated cells, indicating that autophagy inhibition enhances BA-induced apoptosis in NSCLC cells. In line with the cleavage of PARP, flow cytometry analysis also demonstrated that blockage of autophagy with siRNA against ATG5 significantly enhanced BA-induced apoptosis in A549 and H1650 cells (Figure 3C). Collectively, BA provoked protective autophagy, and autophagy blockage enhanced BA-induced apoptosis in NSCLC cells.

### BA Activates the MAPK Pathway and has a Synergistic Anticancer Effect with ERKi (U0126) on NSCLC Cells

**FIGURE 4:**
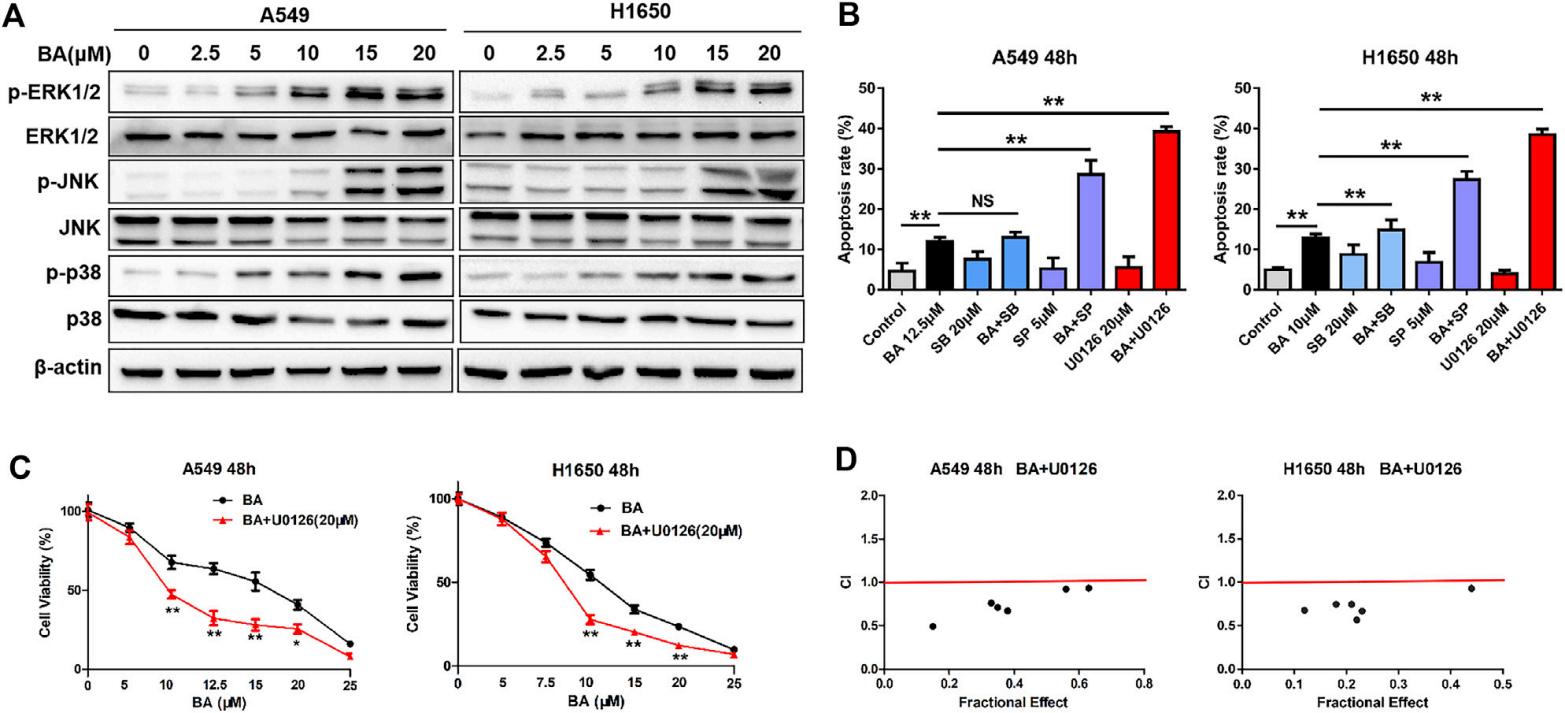
BA activates the MAPK pathway and has a synergistic anticancer effect with U0126 on NSCLC cells. **(A)** A549 and H1650 cells were treated with increasing concentrations of BA for 48 h, and the expressions of phosphorylated and total ERK1/2, JNK, and p38 were detected using Western blots. **(B)** A549 and H1650 cells were treated with the indicated concentrations of BA, or in combination with the indicated inhibitors for 48 h, and cell apoptosis was analyzed by flow cytometry. **(C)** A549 and H1650 cells were treated with increasing concentrations of BA alone or in combination with 20 μM U0126 for 48 h, and cell viability was measured using the MTT assay. **(D)** The synergistic cytotoxicity of BA and U0126 was quantitatively analyzed by the combination index. *p < 0.05, **p < 0.01. ns, no significant difference.

Reports have indicated that the mitogen-activated protein kinases (MAPKs) have a dual role, acting as activators or inhibitors, in apoptosis (Peluso et al., 2019; Yue and Lopez, 2020). Thus, we examined the phosphorylation status of ERK, p38, and JNK proteins using Western blots. As shown in Figure 4A, BA increased the expressions of p-ERK, p-JNK, and p-p38 in A549 and H1650 cells in a dose-dependent fashion, with no effect on these total proteins, implying that BA activated the MAPK pathway. To determine the role of the activated MAPK pathway in BA-induced apoptosis, we employed U0126 (ERK inhibitor), SP600125 (JNK inhibitor), or SB203580 (p38 inhibitor), to co-incubate NSCLC cells with BA. As shown in Figure 4B, these inhibitors alone did not induce apoptosis, but U0126 or SP600125 strikingly enhanced BA-induced apoptosis in A549 and H1650 cells, whereas SB203580 failed to yield any such enhancement. Importantly, the effect of U0126 on the BA-induced apoptosis was superior to that of SP600125. These results suggest that the BA-activated MAPK pathway antagonized BA-induced apoptosis in NSCLC cells.

Drug combination is a common strategy for cancer treatment. Based on the above results, we hypothesized that BA and U0126 have a synergistic anticancer effect on NSCLC cells. As expected, our results showed that U0126 significantly enhanced the cytotoxic effect of BA (Figure 4C), suggesting that BA and U0126 have a synergistic anticancer effect, while U0126 alone had little anticancer activity on NSCLC cells (data not present). Next, the combination index (CI) was utilized to confirm whether there is an additive or synergistic effect between BA and U0126. The result shows that U0126 (20μM) yielded a maximal CI of 0.67 and 0.69 when the dose of BA was 12.5 and 10 μM for A549 and H1650 cells, respectively (Figure 4D), suggesting that U0126 and BA have a highly synergistic anticancer effect on NSCLC cells.

### BA in Combination with U0126 Increases Cytoprotective Autophagy and Activates the AKT Pathway

**FIGURE 5:**
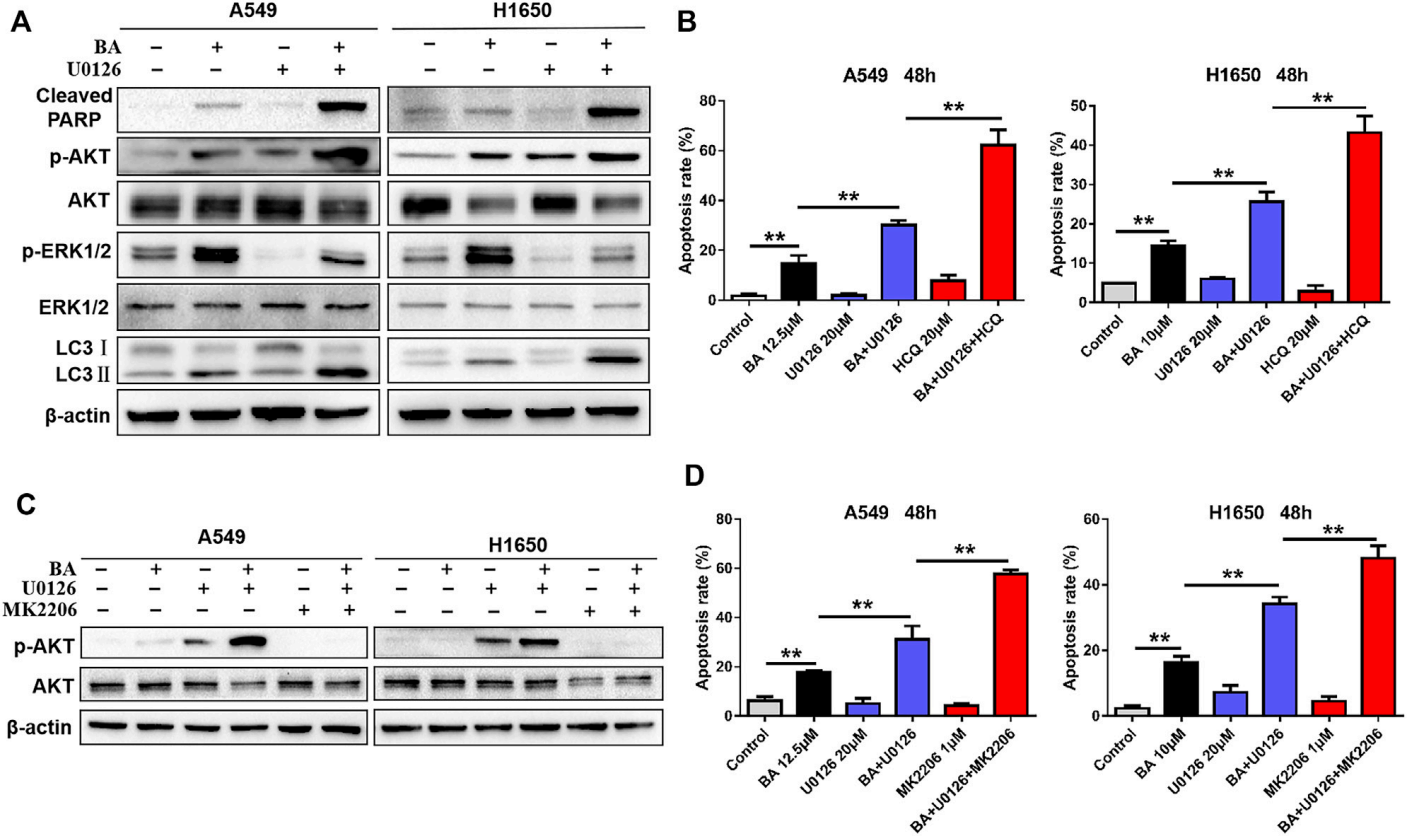
Combined treatment of BA and U0126 activates the AKT pathway and autophagy. **(A)** A549 and H1650 cells were treated with BA alone, U0126 alone, or in combination for 48 h, and the levels of AKT, p-AKT, LC3, cleaved PARP, ERK, and p-ERK1/2 were determined using Western blots. **(B)** A549 and H1650 cells were treated with BA alone, U0126 alone, HCQ alone, or the indicated combinations for 48 h, and apoptosis was measured by flow cytometry. **(C)** A549 and H1650 cells were treated with the indicated chemicals alone or in combination for 48 h, and the levels of AKT and p-AKT were evaluated by Western blots. **(D)** A549 and H1650 cells were treated with the indicated chemicals alone or combination of the chemicals for 48 h, and apoptosis was detected using flow cytometry. **p < 0.01.

To explore the mechanisms by which BA exhibited synergistic anticancer activity with U0126, we examined expressions of related proteins by Western blots when NSCLC cells were treated with combination of BA and U0126. As expected, U0126 significantly attenuated BA-induced ERK activation and enhanced the level of cleaved PARP in A549 and H1650 cells (Figure 5A), consistent with the result shown in Figure 4B. Surprisedly, however, we found that the combined treatment of BA and U0126 also resulted in a strong increase in the level of LC3-II (Figure 5A), indicating that ERK inhibition significantly enhances the BA-induced autophagy. Given that BA treatment induced cytoprotective autophagy in NSCLC cells as demonstrated in the above experiments, we then investigated if the increased autophagy by U0126 leads to cell survival. As shown in Figure 5B, the autophagy inhibitor, HCQ, markedly augmented BA-induced apoptosis in A549 and H1650 cells, whereas HCQ alone had no obvious effect on apoptosis in NSCLC cells. Thus, BA in combination with U0126-induced autophagy protected NSCLC cells against this combination treatment.

In NSCLC cells treated with BA and U0126, we noticed that BA or U0126 alone not only activates AKT but also has a synergistic activation of AKT (Figure 5A). It is well known that AKT always acts as an oncogene in many types of cancer, and ERK can cross talk with AKT (Dent, 2014). Here, U0126 treatment remarkably inhibited ERK but led to a significant increase of phosphorylation of AKT in A549 and H1650 cells, suggesting that ERK inhibition induced feedback activation of AKT. Then, an AKT specific inhibitor, MK2206 was used to co-treat NSCLC cells with BA and U0126, and immunoblotting revealed that MK2206 clearly abolished the activation of AKT caused by U0126 and BA in A549 and H1650 cells, respectively (Figure 5C). Importantly, MK2206 significantly increased the proapoptotic efficacy of BA and U0126 in A549 and H1650 cells, respectively (Figure 5D). Taken together, combined treatment of BA and U0126 activated the AKT pathway that served as a pro-survival role in NSCLC cells.

### Trametinib Increases BA-Induced Cell Cycle Arrest and Apoptosis

**FIGURE 6:**
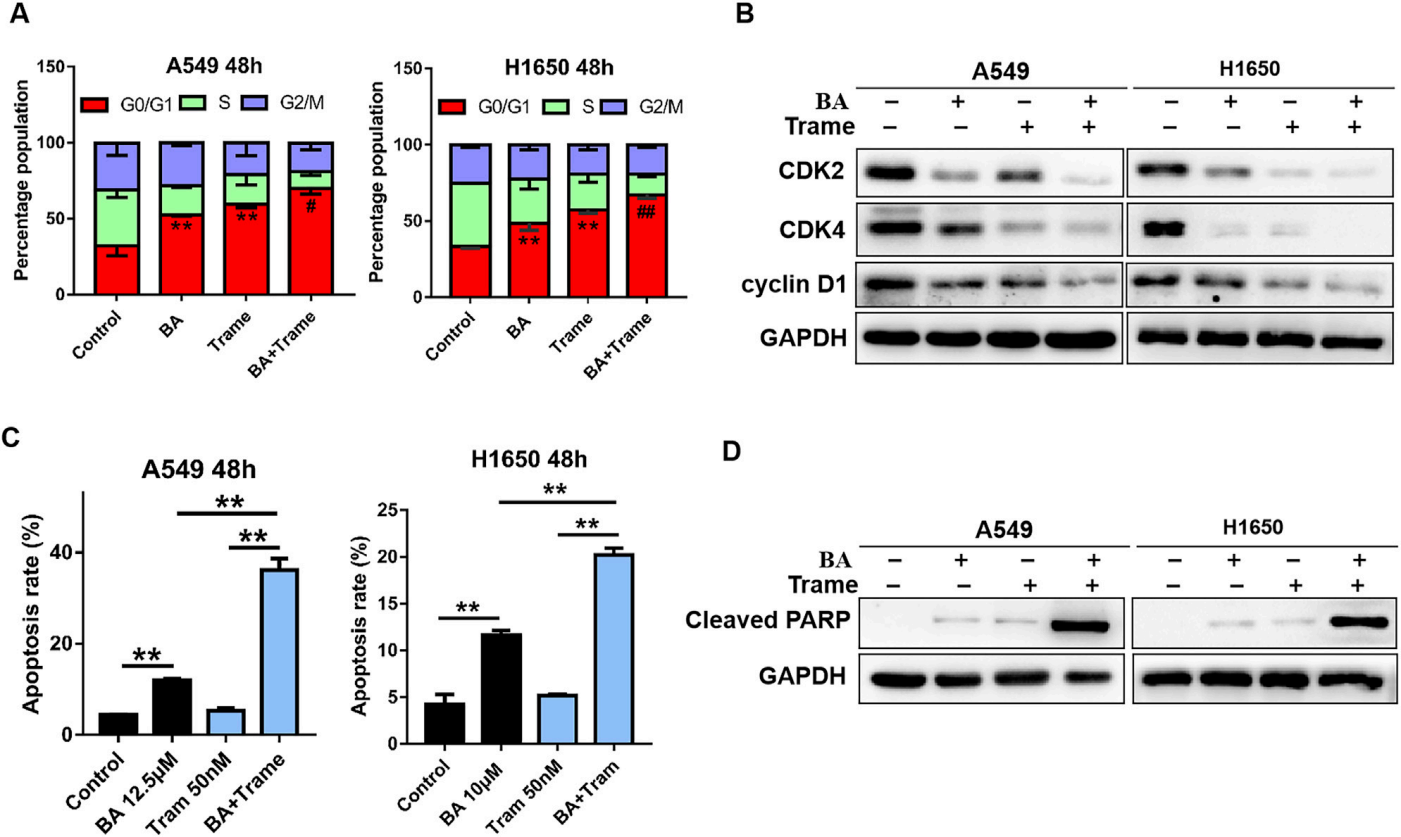
Trametinib increases BA-induced cycle arrest and apoptosis in A549 cells. **(A)** A549 and H1650 cells were treated with 12.5 μM BA, 50 nM trametinib, or in combination for 48 h, and cells were harvested for cell cycle analysis by flow cytometry. **(B)** A549 and H1650 cells were treated with 12.5 μM BA, or 50nM trametinib, or in combination for 48 h, and the expressions of cyclin D1, CDK2, and CDK4 were evaluated by Western blots. **(C)** A549 and H1650 cells were treated with 12.5 μM BA, or 50 nM trametinib, or in combination for 48 h, and apoptosis was detected using flow cytometry. **(D)** A549 and H1650 cells were treated with 12.5 μM BA, or 50nM trametinib, or in combination for 48 h, and the expression of cleaved PARP was evaluated using Western blots. #*p* < 0.05, ##*p* < 0.01 vs BA or trametinib alone, **p < 0.01.

Although U0126 exhibits a highly synergistic inhibition on NSCLC cells with BA, indicating that the ERK pathway is a potential combinational therapeutic target, it is not a clinical antitumor drug. Therefore, safer and more effective ERKi are needed to optimize the use of BA. Recently, the FDA-approved trametinib and MEK162 were used as another ERKi for cancer therapy. Thus, we employed trametinib to replace U0126 in the next experiments. We treated NSCLC cells with BA or trametinib, or in combination, and a flow cytometer was used to test cell cycle and apoptosis. Indeed, increased G0/G1 phase arrest was observed in A549 and H1650 cells upon treatment with BA or trametinib alone, and the combination treatment (BA plus trametinib) had synergetic efficacy in cycle arrest (Figure 6A). Moreover, immunoblotting revealed that BA or trametinib alone reduced the levels of CDK2, CDK4, and cyclin D1, and the combination of both induced a synergistic decrease in the levels of those proteins in NSCLC cells (Figure 6B). Furthermore, 50 nM trametinib alone had no striking effect on apoptosis in A549 and H1650 cells, whereas combined treatment of BA and trametinib led to an increased apoptosis in NSCLC cells (Figure 6C). Accordingly, the combined treatment efficiently upregulated the apoptosis marker, cleaved PARP (Figure 6D). Thus, trametinib increases BA-induced cell cycle arrest and apoptosis in NSCLC cells.

### Combined Triple-Drug Treatment Enhances Cytotoxic Activity in NSCLC Cells

**FIGURE 7:**
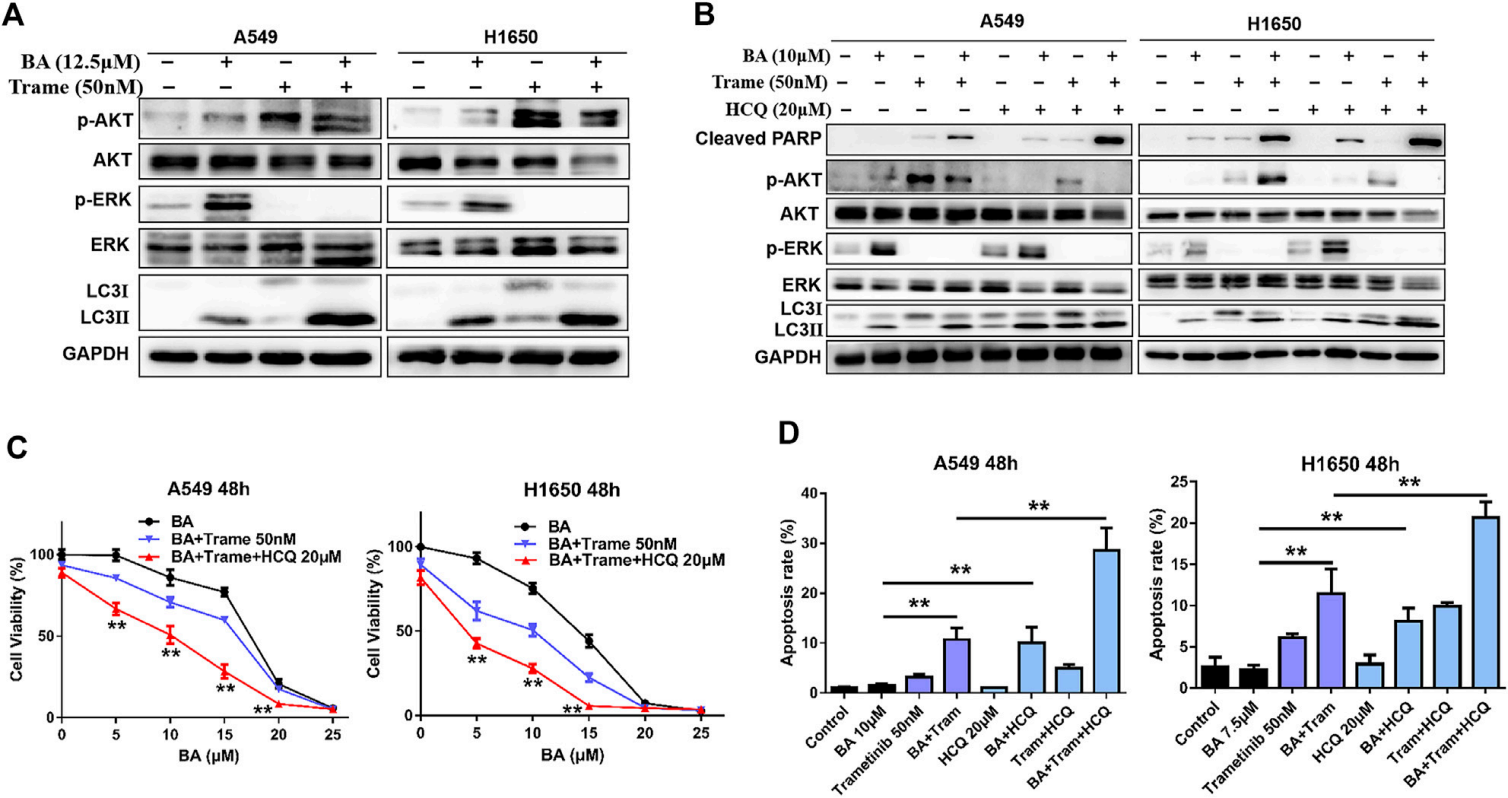
Triple-drug therapy was the most effective for lung cancer cells *in vitro*. **(A)** A549 and H1650 cells were treated with 12.5 μM BA, 50 nM trametinib, or in combination for 48 h, and the levels of AKT, p-AKT, LC3, cleaved PARP, ERK, and p-ERK1/2 were determined by Western blots. **(B)** A549 and H1650 cells were treated with indicated chemicals alone or in combination for 48 h, and the levels of AKT, p-AKT, LC3, cleaved PARP, ERK, and p-ERK1/2 were determined by Western blots. **(C)** A549 and H1650 cells were treated with indicated chemicals alone, or in combination for 48 h, and cell viability was measured by the MTT assay. **(D)** A549 and H1650 cells were treated with indicated chemicals alone, or in combination for 48 h, and cell apoptosis was detected using flow cytometry. **p < 0.01.

**FIGURE 8:**
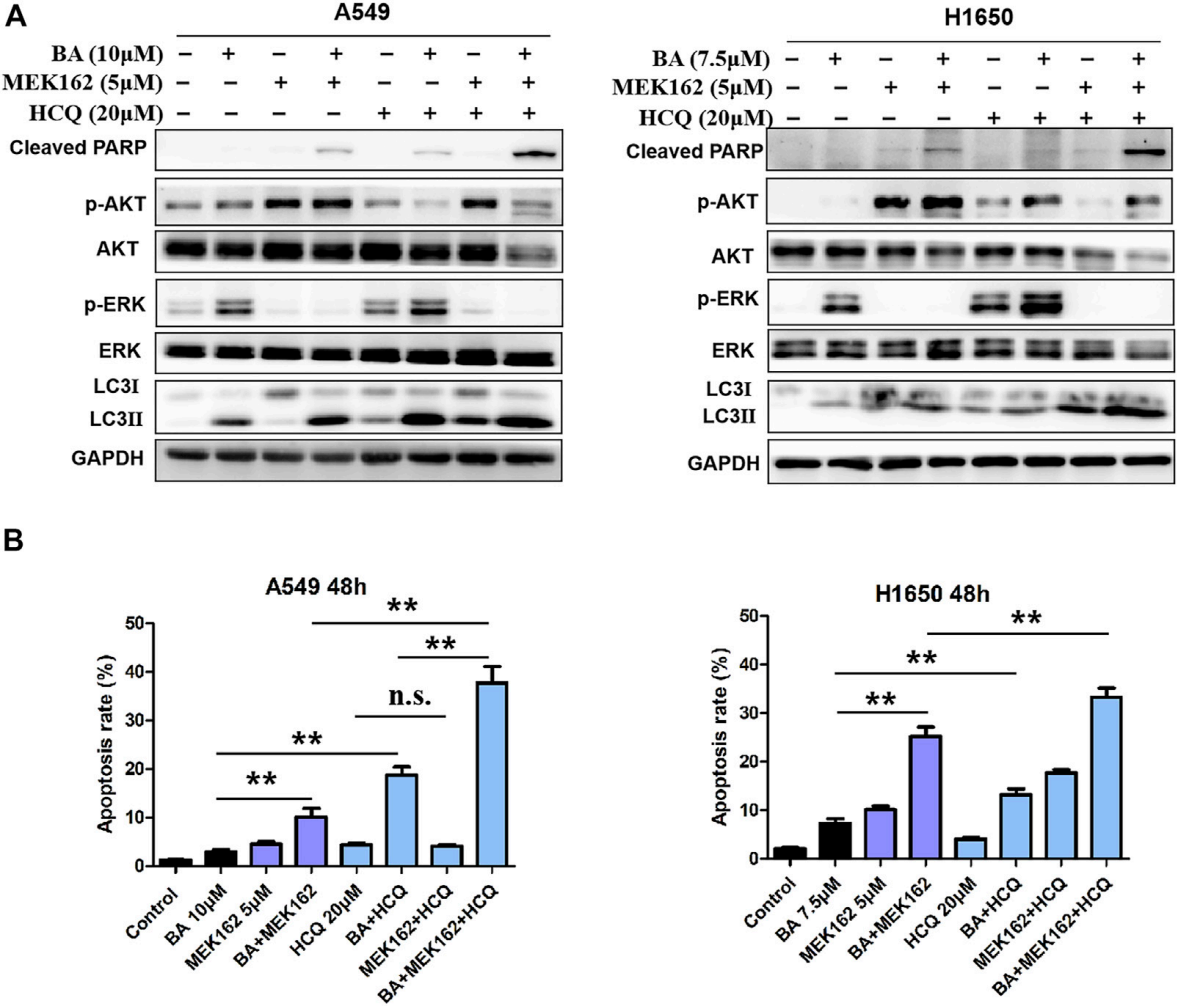
Triple-drug therapy enhances therapeutic activity *in vitro*. **(A)** A549 and H1650 cells were treated with indicated concentrations of BA alone or in combination with 5 μM MEK162, or in combination with MEK162 plus 20 μM HCQ for 48 h, and the levels of AKT, p-AKT, LC3, cleaved PARP, ERK, and p-ERK1/2 were determined by Western blots. **(B)** A549 and H1650 cells were treated with indicated chemicals alone or in combination for 48 h, and apoptosis was detected using flow cytometry. **p < 0.01. ns, no significant difference.

Then we examined whether the efficacy of trametinib or MEK162 is similar to that of U0126 that has feedback activation with AKT and activates autophagy. As shown in Figure 7A, trametinib fully abrogated BA-induced ERK activation in A549 and H1650 cells but also increased the levels of AKT phosphorylation and autophagy (LC3II protein), which attenuated antitumor effect of BA. Therefore, combining HCQ with BA plus trametinib can enhance the antitumor effect on NSCLC cells as combined treatment of AKT or an autophagy inhibitor, BA and U0126, in the above experiments. As expected, BA combined with trametinib increased the proliferation-inhibitive effect on NSCLC cells, and this efficacy was enhanced by the addition of HCQ (Figure 7C). Furthermore, HCQ inhibited autophagosome fusion with lysosome, leading to an increasing level of autophagy marker LC3-II as shown by the Western blot (Figures 7B and 8A). Interestingly, dual inhibition of ERK (trametinib or MEK162) and autophagy (HCQ) abated the activation of AKT in A549 and H1650 cells (Figures 7A and 8A), indicating that blockage of autophagy attenuated the compensatory activation of AKT by ERK inhibition. Compared with cells treated with BA in combination with trametinib or MEK162, triple-drug combination (BA, trametinib or MEK162, and HCQ) treatment resulted in increased expression of the apoptosis marker, cleaved PARP (Figures 7A and 8A), which was consistent with the apoptosis generated by the same combination treatment in A549 and H1650 cells as shown by the flow cytometry assay (Figures 7D and 8B). Taken together, our results suggest that triple-drug combination treatment was superior to either single drug or two drugs *in vitro*.

### Triple-Drug Therapy Inhibits Tumor Growth in the Xenograft Model

**FIGURE 9:**
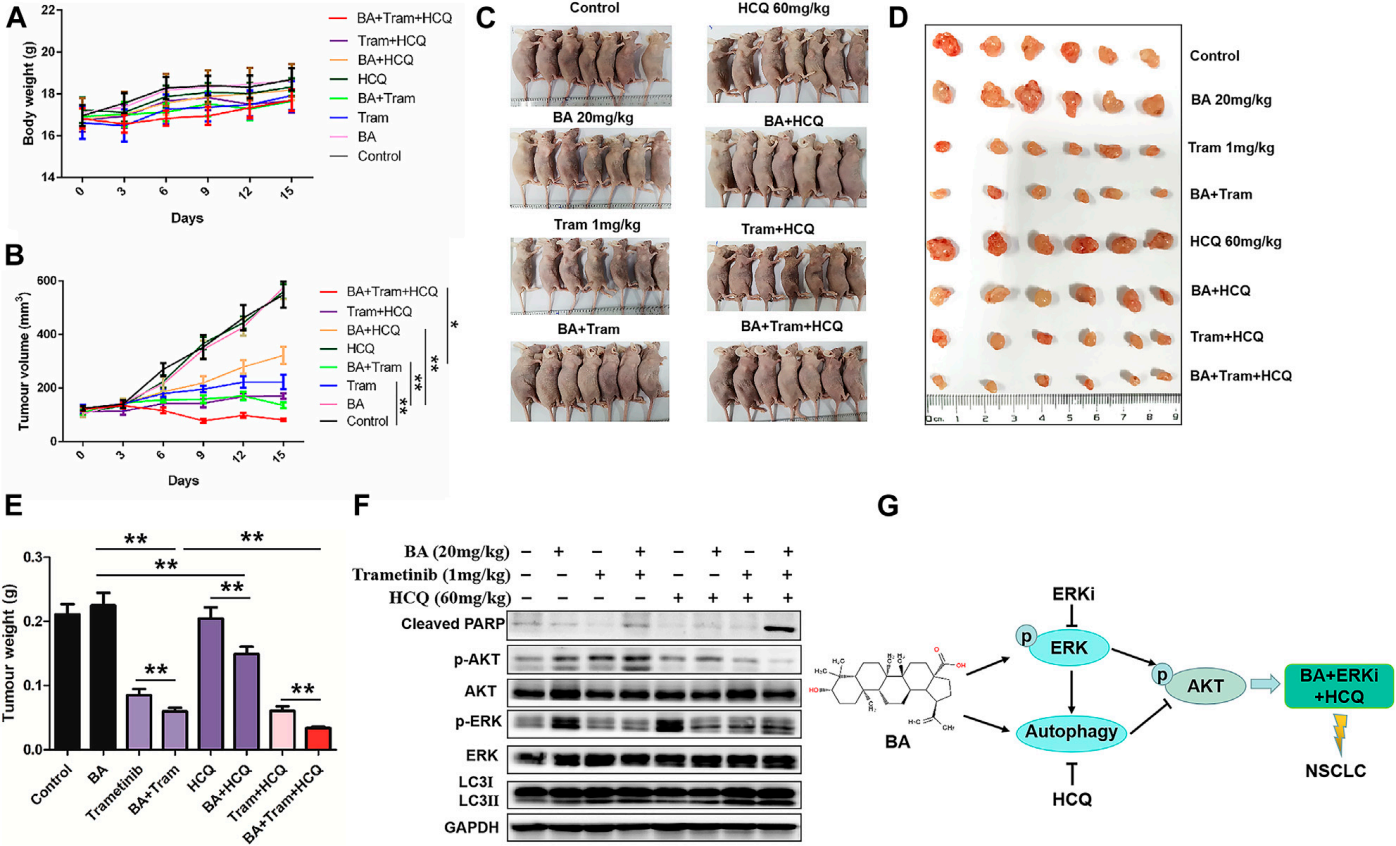
Triple-drug therapy was the most effective for NSCLC *in vivo*. A549 xenograft mice were treated with the vehicle control, BA (20 mg/kg), trametinib (1 mg/kg), BA + trametinib, HCQ (60 mg/kg), BA + HCQ, trametinib + HCQ, and BA + trametinib + HCQ. Mouse body weights **(A)** and tumor sizes **(B)** were measured as indicated. **(C)** After treatment for 15 days, mice were humanely euthanized, and images of mice from each group (*n* = 7) were shown. **(D)** Tumor photographs dissected out from the mice. **(E)** Average tumor weight of each group (*n* = 7). **(F)** The levels of AKT, p-AKT, LC3, cleaved PARP, ERK, and p-ERK1/2 in mice tumor were detected by Western blots. **(G)** Schematic illustration showing the basis for the combinatorial application. *p < 0.05, **p < 0.01.

To further evaluate the efficacy of the combined treatment *in vivo*, we generated a xenograft mouse model with A540 cells as described in Materials and Methods. In the experiment, mice treated with any combination therapy showed no significant weight loss when compared with the control group (Figure 9A), suggesting that the toxicity of these therapies was tolerant. As shown in Figures 9B–E, there was no significant change of tumor growth after either BA or HCQ treatment alone in comparison with control mice; however, a notable decrease of tumor growth was observed in the combination of BA- and HCQ-treated mice, which was consistent with *in vitro* cell experiments (Figure 3A); furthermore, a single dose of 1 mg/kg trametinib substantially reduced tumor burdens, and this efficacy was strikingly enhanced by BA treatment; most importantly, triple-drug combination (BA and trametinib plus HCQ) had the best therapeutic efficacy in tumor-bearing mice, similar to the observed results *in vitro*. In addition, immunoblotting revealed that BA alone or in combination with trametinib caused an increased expression of LC3-II and decreased phosphorylation of AKT in the tumor tissues (Figure 9F). Consistently, trametinib reduced BA-induced ERK phosphorylation. As expected, triple-drug combination increased the expression of cleaved PARP, and abrogated BA or combined with trametinib-induced activation of AKT (Figure 9F). Thus, these data demonstrate that triple-drug combination can highly suppress tumor growth in the xenograft model.

## Discussion

Our study has identified that co-treatment of BA and ERKi or in combination with autophagy inhibitor resulted in dramatically synergistic anticancer effect in NSCLC cells. Of note, BA alone or combined with ERKi induced protective autophagy, while autophagy blockage augmented these treatment-induced cell deaths. In addition, the combined therapy of BA and ERKi appeared to activate the AKT pathway in NSCLC cells, and combined targeting inhibition of AKT increased the therapeutic efficacy. Importantly, the three-drug combination (BA, ERKi, and HCQ) resulted in superior therapeutic efficacy than single or dual treatments *in vitro* and *in vivo*.

Autophagy still plays an important role in cancer therapy. Defective autophagy can drive healthy cells to undergo malignant transformation, and cancer cells are able to harness autophagy to thrive in response to various adverse conditions (Amaravadi et al., 2019). Evidence indicates that malignant cells are more addicted to autophagy than normal cells and even have “autophagy addiction” (Mowers et al., 2018). However, the role of autophagy in cancer is highly context-dependent. Increasing evidence reveals that activated autophagy can serve as type-II programmed cell death by providing a scaffold for the cell death process (Doherty and Baehrecke, 2018). Consistent with this, we previously reported that scutellarin combined with cisplatin induced autophagic cell death, and inhibition of autophagy attenuated the synergism of this combination (Sun et al., 2018). By contrast, extensive reports indicate that autophagy can be employed by cancer cells to resist therapeutic challenges, which is consistently validated by this study. BA alone or in combination with ERKi triggered protective autophagy, and inhibition of autophagy allowed NSCLC cells to undergo apoptosis. *In vivo*, HCQ addition further delayed tumor growth in mice treated by BA alone, or in combination with trametinib. Thus, BA or BA plus ERKi, in combination with autophagy inhibition, has considerable potential as a therapeutic approach for NSCLC.

It is known that ERK is a pivotal downstream effector of Ras/Raf/ERK signaling, which is frequently mutated in lung adenocarcinoma (Samatar and Poulikakos, 2014). Thus, inhibitors targeting ERK have remarkable clinical anticancer activity. However, numerous studies demonstrate that constitutive activation of ERK plays an important role in cell death, making it difficult to define its exact function. For example, fisetin, a natural flavonoid, suppresses the proliferation and metastasis of renal cell carcinoma by activating ERK, and such inhibitory effect is reversed by ERK inhibition (Hsieh et al., 2019). Moreover, hyperactivation of the ERK pathway is deleterious to K-Ras mutation-driven lung cancer cells (Unni et al., 2018). Here, BA treatment caused activation of the ERK pathway in NSCLC cells, which raises our great interest to reveal its context-specific role. U0126, a specific MEK/ERK inhibitor, was selectively used to restrain ERK. It has been reported that U0126 enhances the cytotoxicity of radiotherapy in rhabdomyosarcoma cells through suppression of DNA repair signals (Marampon et al., 2011). In addition, U0126 increases the anticancer activity of combretastatin A4, depending on MEK inhibition (Quan et al., 2009). Our data show that U0126 in combination with BA resulted in a synergistic anticancer effect, suggesting that activated ERK by BA displayed oncogenic activity. Interestingly, this combined therapy was not consistent with previous report that BA-induced apoptosis was antagonized by the addition of U0126 (Rieber and Rieber, 2006).

Although U0126 can enhance BA-induced cytotoxicity in NSCLC cells, in fact, no clinical trials have been conducted on U0126 due to its pharmacologic limitations *in vivo*. Trametinib, another potent ERKi, has been approved by the FDA as a single agent or in combination with dabrafenib for treating unresectable or metastatic melanoma (Lian et al., 2019). Binimetinib, also known as MEK162, received the first approval in 2018 for use in combination with encorafenib to treat advanced melanoma (Shirley, 2018). Similar to U0126, trametinib or MEK162 enhanced the therapeutic activity of BA, suggesting that the combination will offer clinical benefits. In addition, the inhibitory efficacy of trametinib or MEK162 on ERK was highly superior to that of U0126. Therefore, we hypothesize that ERK inhibition would likewise synergize with other cytotoxic chemotherapy or targeted agents that are capable of activating ERK. For instance, afatinib increases the phosphorylation of ERK in head and neck cancer cells, and inhibitors targeting ERK lead to synergistic anticancer effect with afatinib (Lin et al., 2019). Consistently, ERK activation was sufficient to confer resistance to cisplatin in NSCLC cells *via* upregulation of Bcl-2 (Wu et al., 2013). Thus, our study provides the basis for rational combination of ERKi with cytotoxic agents that can activate the ERK pathway.

Nevertheless, a second potential limitation of ERKi is that ERK inhibition leads to the compensatory activation of PI3K/AKT (Chen et al., 2017; Tsubaki et al., 2019). Similar to ERK, the AKT pathway is a central regulator of cell survival, differentiation, and metabolism (Mayer and Arteaga, 2016). Accumulating evidence reveals that AKT is dysregulated in almost all cancers, and thus AKT inhibition has the potential to therapeutic response to cancer (Hoxhaj and Manning, 2019). As such, co-inhibition of ERK and AKT, rather than either, might be an opportunity for improved therapeutic efficacy. For example, co-treatment of both inhibitors, LY294002 and U0126, yields a synergistic antitumor effect in triple-negative breast cancer (He et al., 2019). In line with this, several clinical trials have been demonstrated that dual inhibition of PI3K/AKT and the Ras/ERK pathway has a dramatically better outcome (Cao et al., 2019). Here, our results show that blockade of ERK was responsible for the increased phosphorylation of AKT. ERKi alone or in combination with BA had the ability to phosphorylate AKT. As such, targeting compensatory AKT activation and silencing of AKT by its specific inhibitor, MK2206, could significantly enhance the susceptibility of NSCLC cells to the combined treatment. Encouragingly, inhibition of autophagy by HCQ not only inhibited cytoprotective autophagy but also suppressed the AKT pathway in NSCLC cells. The mechanism behind this phenomenon is unclear, and further studies on the mechanism are guaranteed.

Overall, our results serve as the basis for the combinatorial application of two or more anticancer drugs (Figure 9G). First, although BA in combination with ERKi has a synergistic antitumor activity, both of them induce cytoprotective autophagy and AKT activation, which limits its efficacy. Second, the three-drug combination (BA, ERKi, and HCQ) resulted in superior therapeutic efficacy than single or dual treatments *in vitro* and *in vivo*. Thus, our study provides the evidence that the drug combination treatment is a therapeutic strategy for NSCLC.

## Data Availability

The raw data supporting the conclusions of this article will be made available by the authors, without undue reservation.
