# Supplementary figures and images for: Combination Treatment With Inhibitors of ERK and Autophagy Enhances Antitumor Activity of Betulinic Acid in Non–small-Cell Lung Cancer In Vivo and In Vitro
Source: Front Pharmacol. 2021 Jun 29;12:684243. doi: 10.3389/fphar.2021.684243 (PMC8275840; doi:10.3389/fphar.2021.684243)

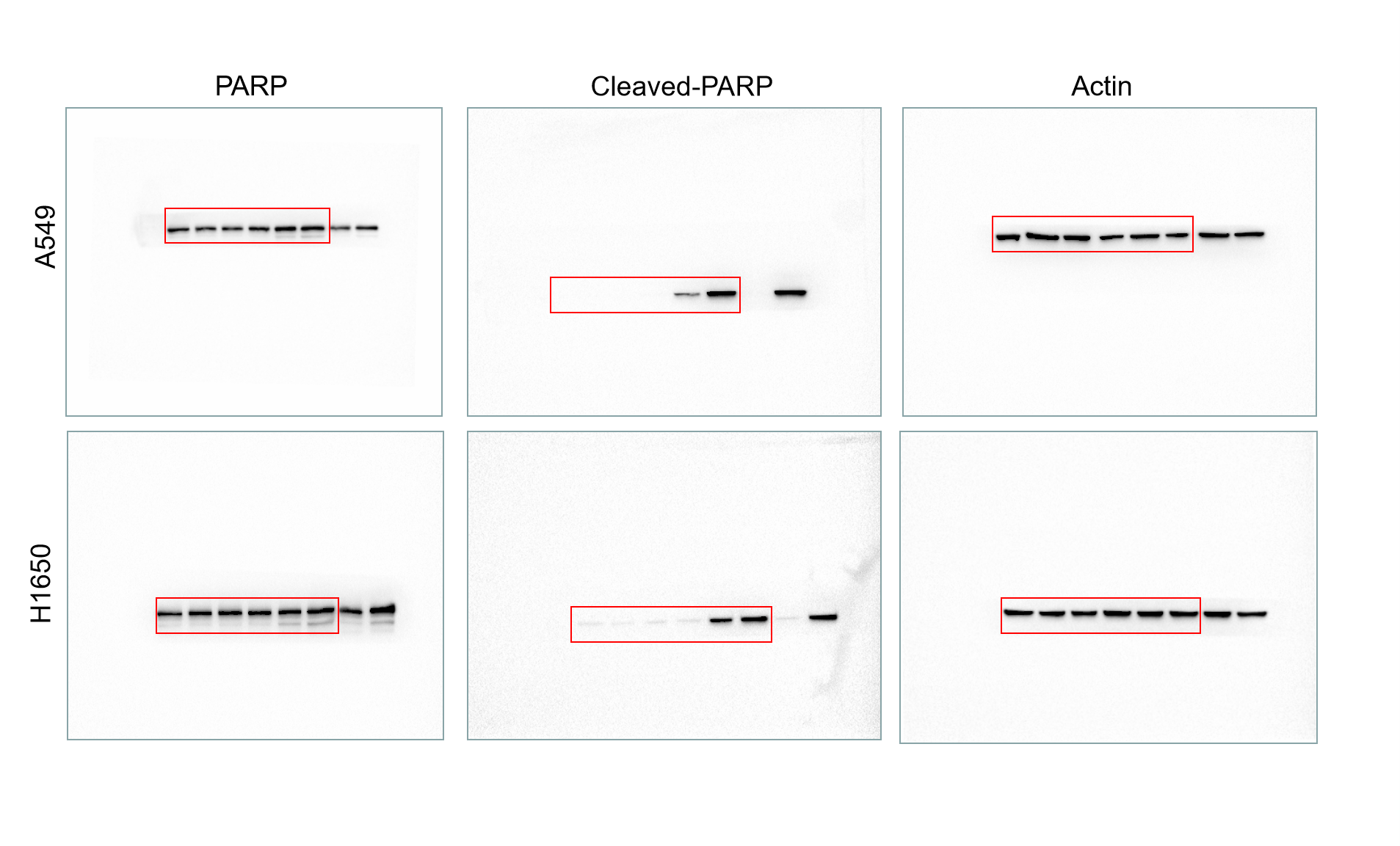

Supplement: Supplementary file 1 [file DataSheet1.ZIP › Original WB data/Supplementary FIG.1.tif]

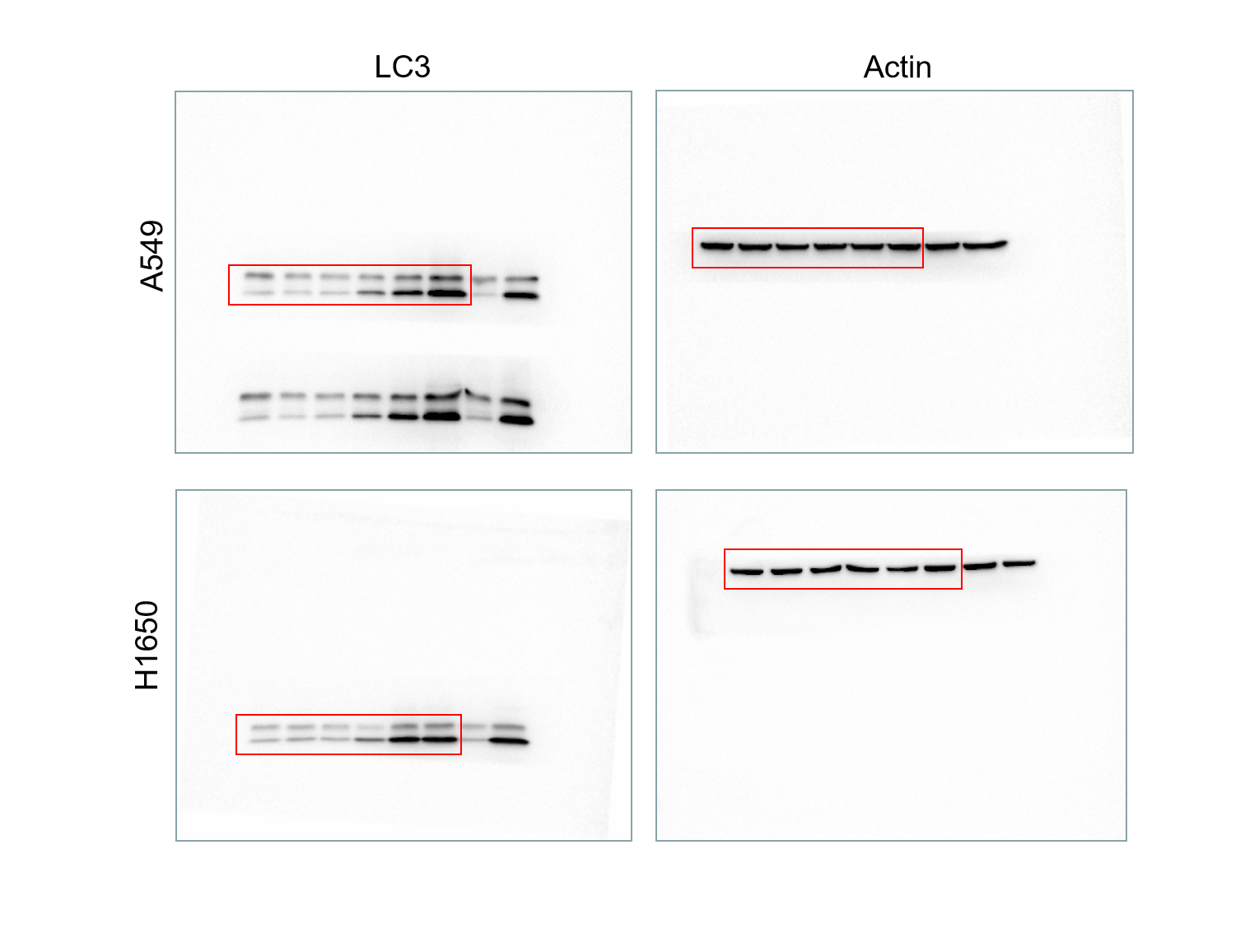

Supplement: Supplementary file 1 [file DataSheet1.ZIP › Original WB data/Supplementary FIG.2.tif]

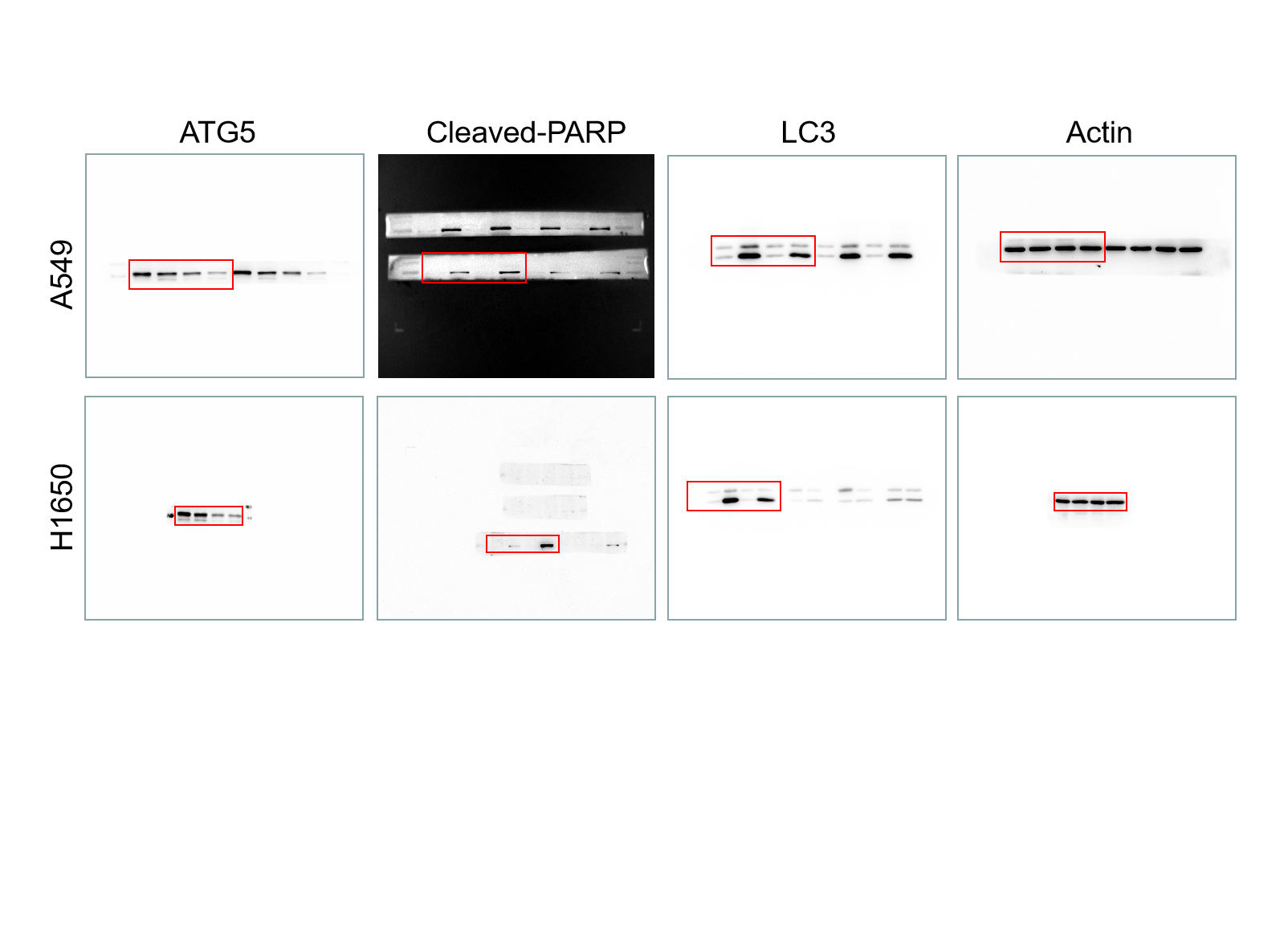

Supplement: Supplementary file 1 [file DataSheet1.ZIP › Original WB data/Supplementary FIG.3.tif]

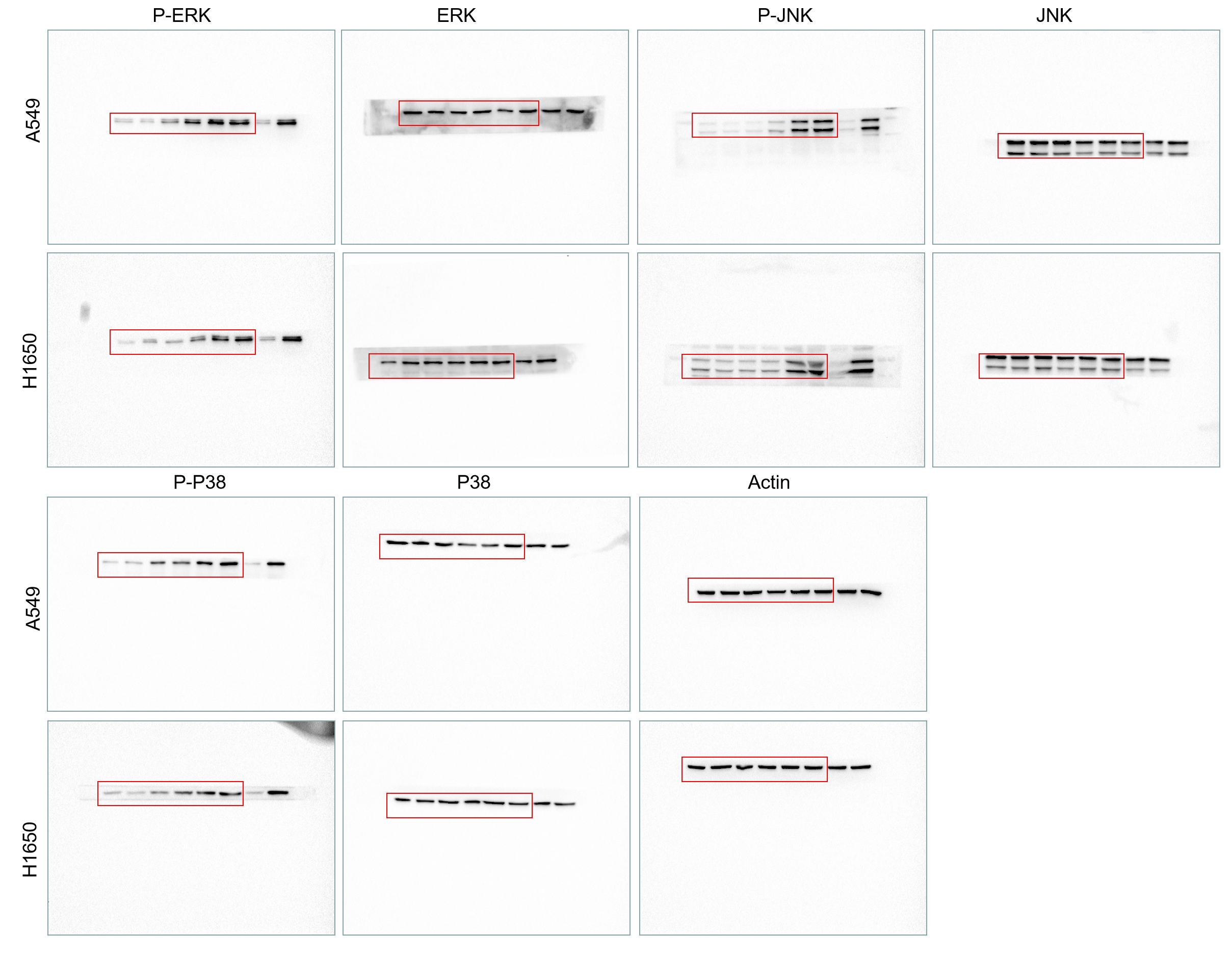

Supplement: Supplementary file 1 [file DataSheet1.ZIP › Original WB data/Supplementary FIG.4.tif]

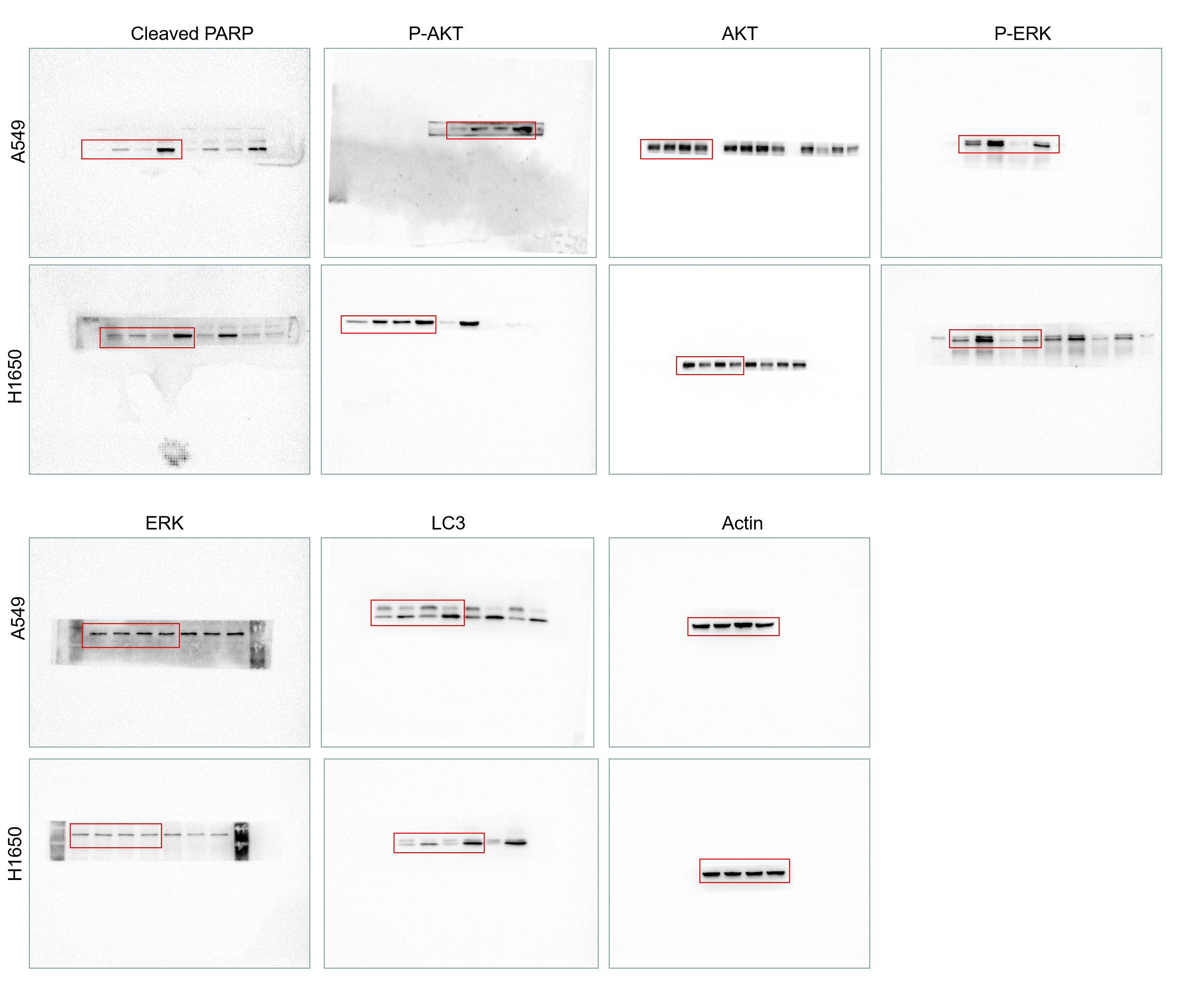

Supplement: Supplementary file 1 [file DataSheet1.ZIP › Original WB data/Supplementary FIG.5A.tif]

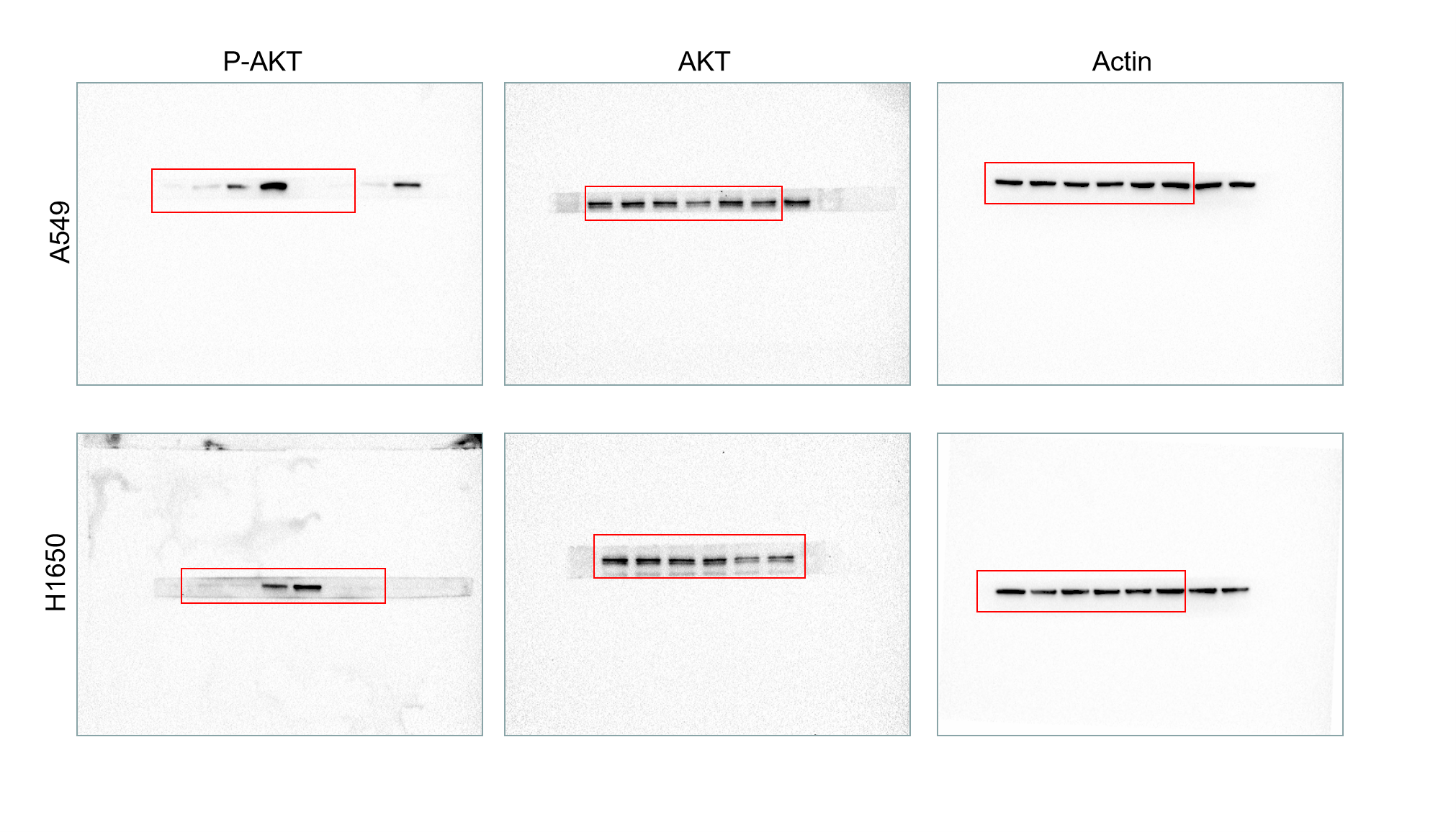

Supplement: Supplementary file 1 [file DataSheet1.ZIP › Original WB data/Supplementary FIG.5C.tif]

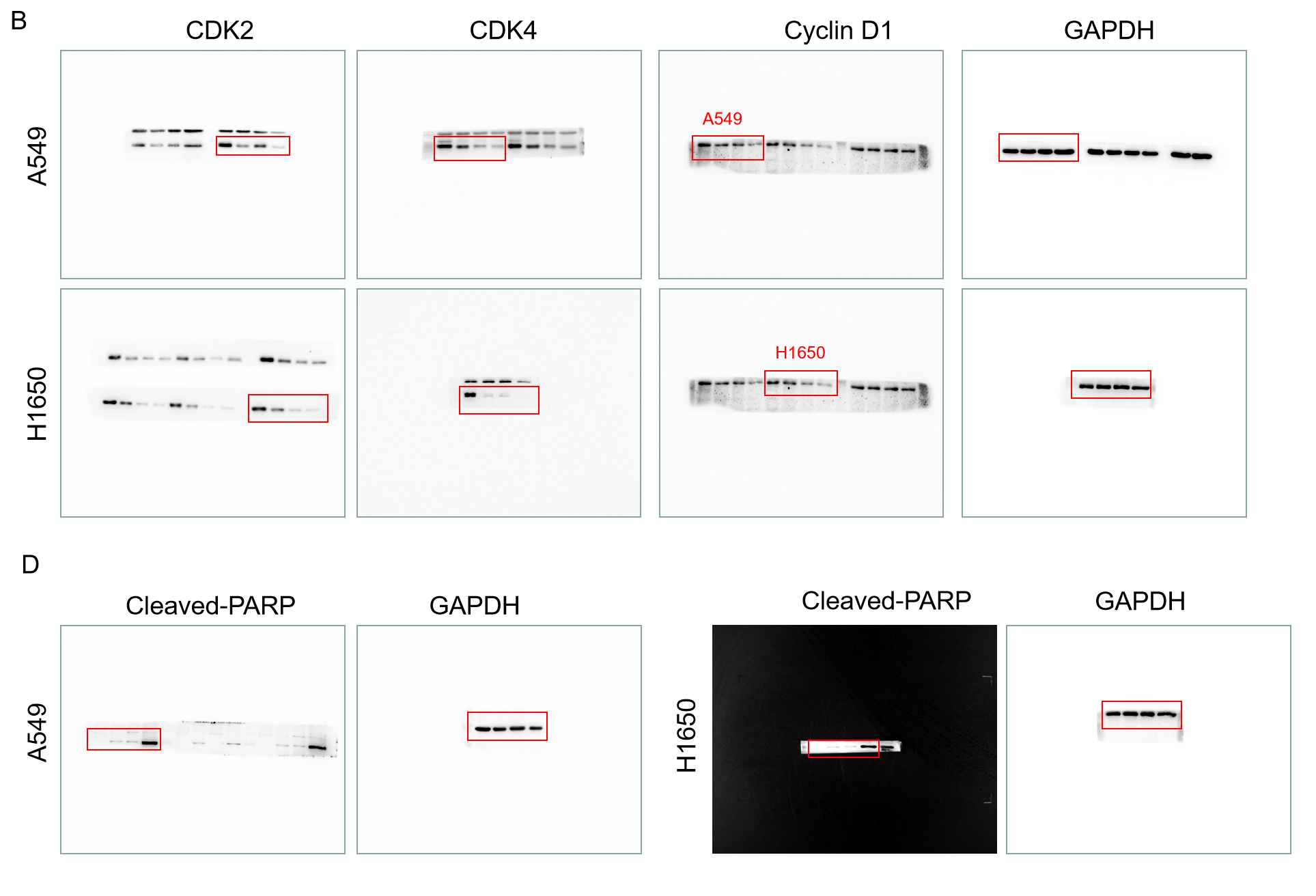

Supplement: Supplementary file 1 [file DataSheet1.ZIP › Original WB data/Supplementary FIG.6.tif]

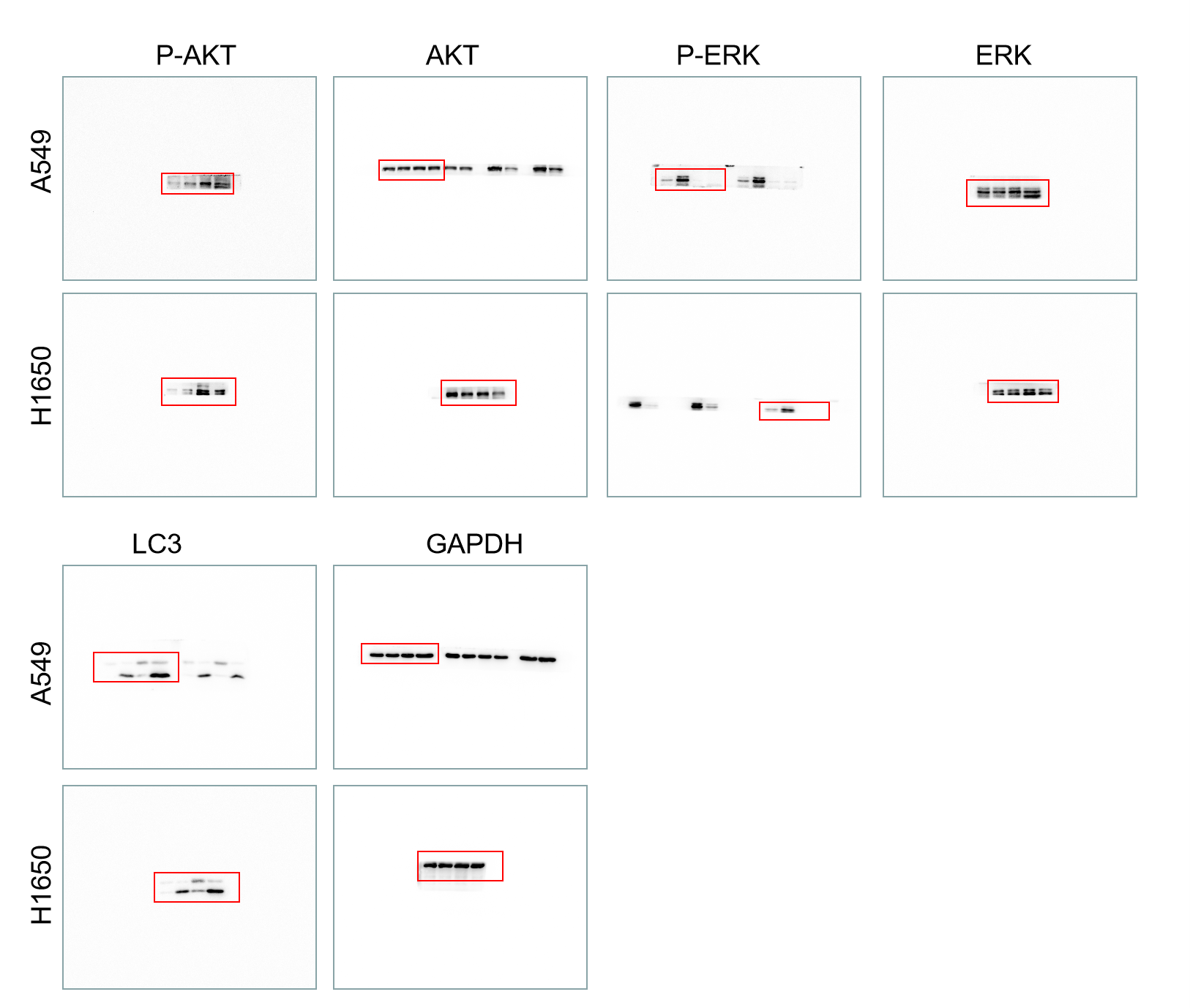

Supplement: Supplementary file 1 [file DataSheet1.ZIP › Original WB data/Supplementary FIG.7A.tif]

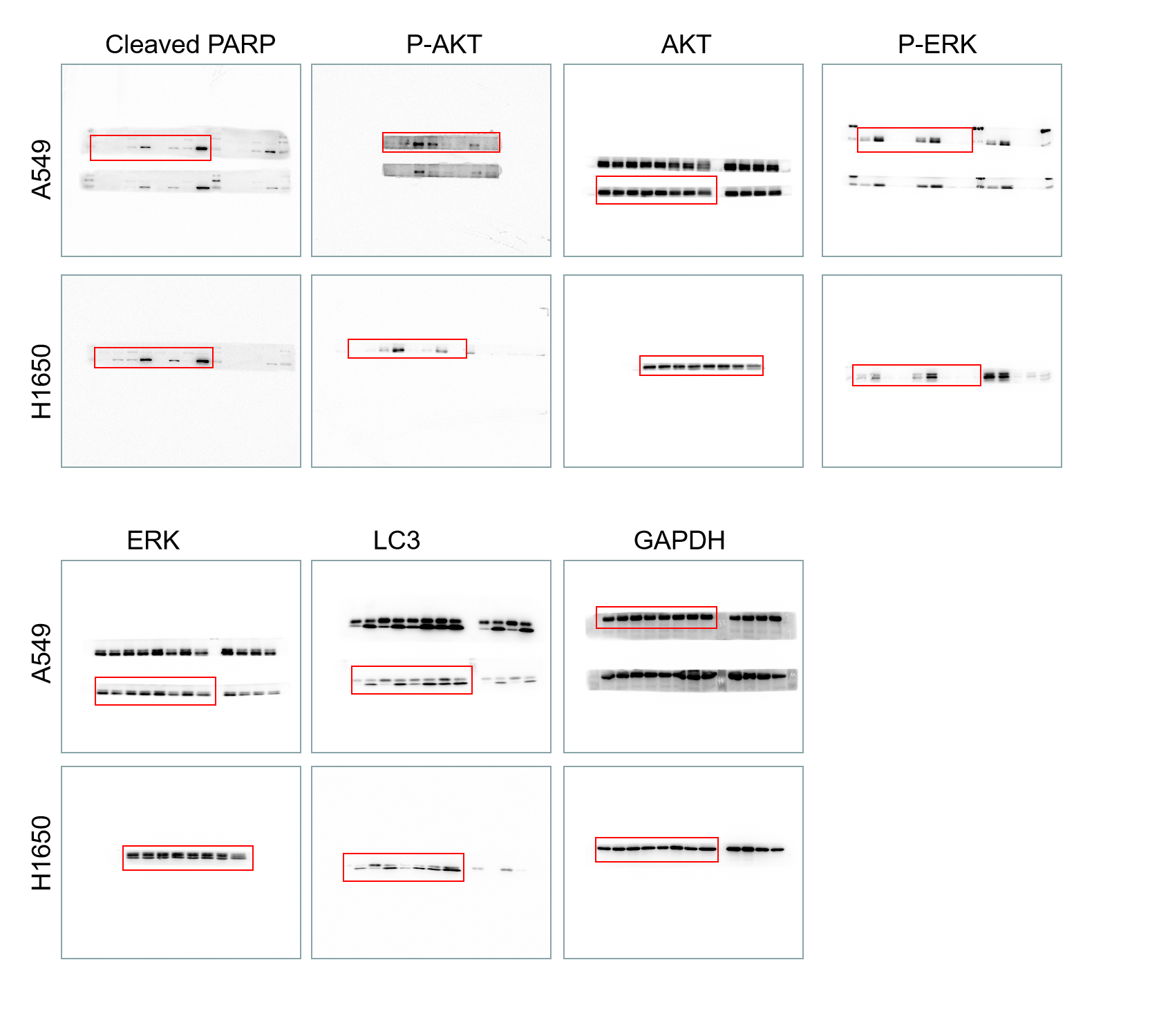

Supplement: Supplementary file 1 [file DataSheet1.ZIP › Original WB data/Supplementary FIG.7B.tif]

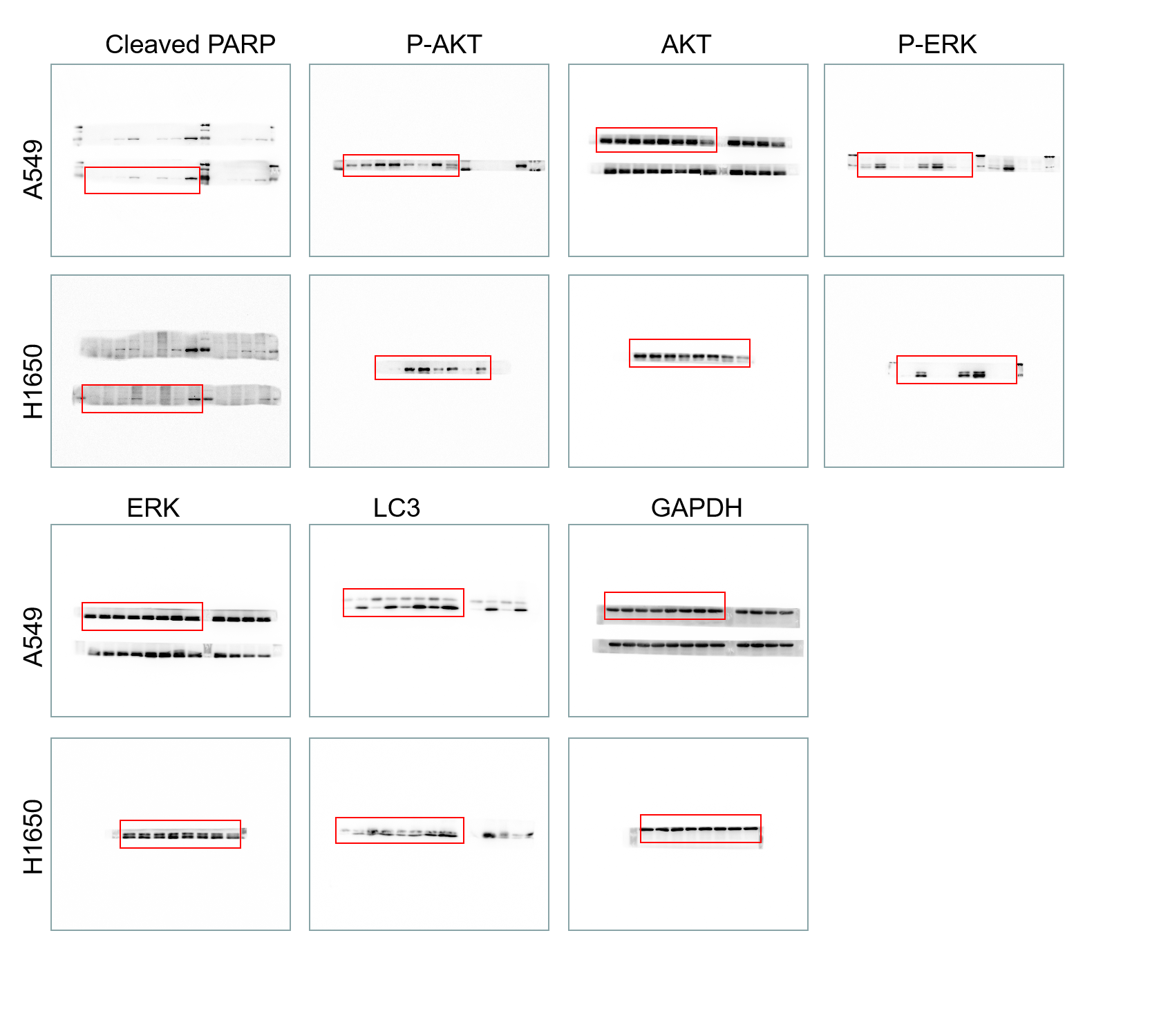

Supplement: Supplementary file 1 [file DataSheet1.ZIP › Original WB data/Supplementary FIG.8.tif]

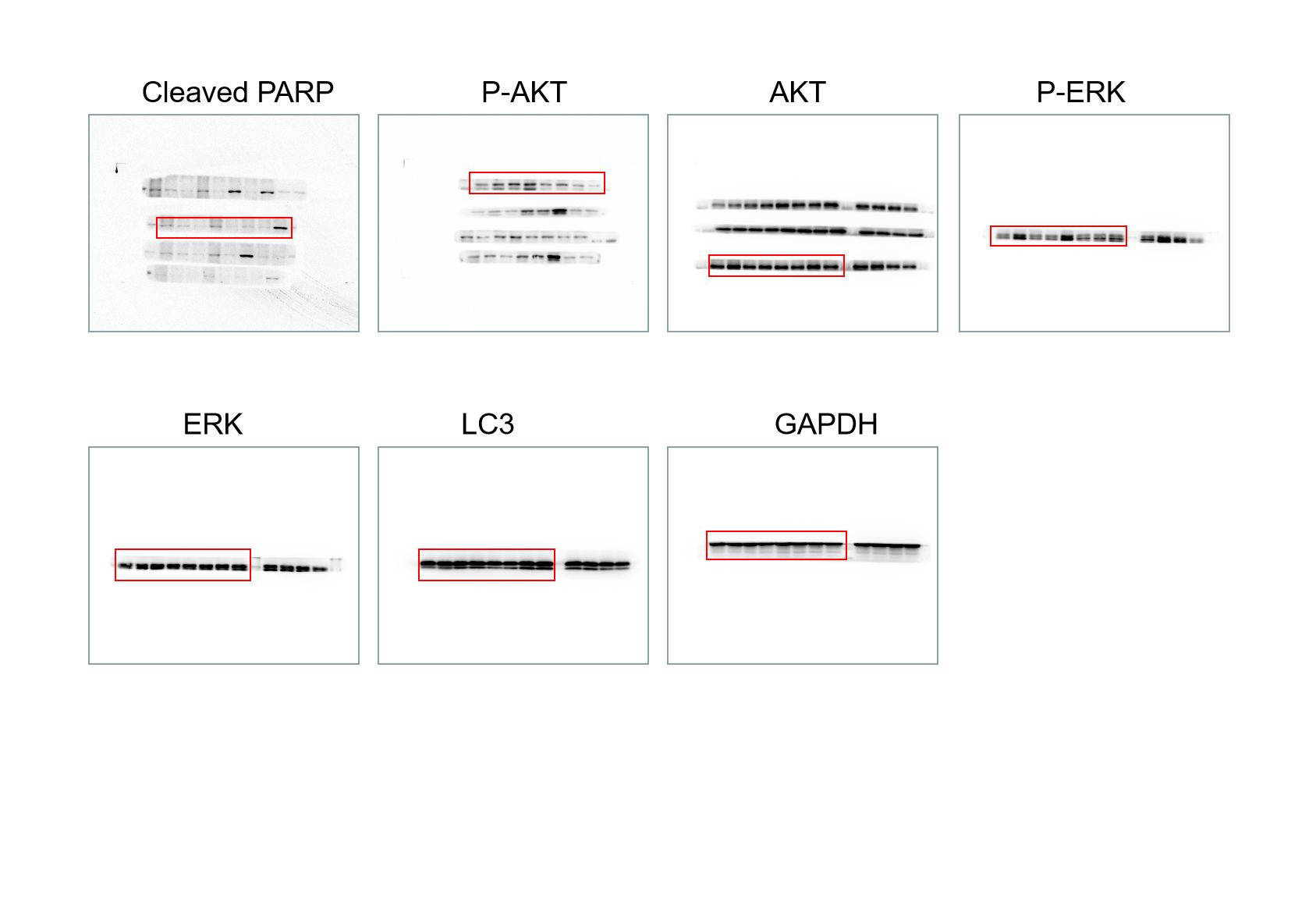

Supplement: Supplementary file 1 [file DataSheet1.ZIP › Original WB data/Supplementary FIG.9.tif]
